# Interleukin-4-Mediated NLRP3 Inflammasome Activation in Microglia Contributes to Allergic Rhinitis via Central Sensitization

**DOI:** 10.34133/research.0897

**Published:** 2025-09-25

**Authors:** Hao Lv, Yunfei Wang, Lu Tan, Yulie Xie, Peiqiang Liu, Mengting Guan, Jianchao Cong, Yu Xu

**Affiliations:** ^1^ Department of Otolaryngology-Head and Neck Surgery, Renmin Hospital of Wuhan University, Wuhan, China.; ^2^ Department of Rhinology and Allergy, Renmin Hospital of Wuhan University, Wuhan, China.; ^3^ Research Institute of Otolaryngology-Head and Neck Surgery, Renmin Hospital of Wuhan University, Wuhan, China.; ^4^ Hubei Province Key Laboratory of Allergy and Immunology, Wuhan, China.

## Abstract

**Background:** The hypersensitive state of the central nervous system, commonly known as central sensitization, presumably drives allergic rhinitis (AR) pathogenesis. However, the involvement of central sensitization in AR and its related mechanisms have rarely been studied. **Methods:** An AR mouse model was induced via ovalbumin treatment. Activation of trigeminal nucleus caudalis (TNC) neurons was assessed by electrophysiological recordings and immunofluorescence staining. The role of TNC neurons in AR was investigated by selectively manipulating them through chemogenetics. The contribution of microglial neuroinflammation to central sensitization and AR was assessed by pharmacologically inhibiting microglial activation. The mechanism of central sensitization in AR was further determined using a microglia–neuron co-culture system. The role of interleukin (IL)-4 in neuroinflammation in AR was explored using in vivo and in vitro methods. **Results:** The intrinsic neuronal excitability and molecular markers of central sensitization were increased within the TNC of AR mice. Chemogenetic inhibition of TNC neurons ameliorated nasal symptoms, histological changes, autonomic dysfunction, and immune imbalance in AR mice. Moreover, AR mice exhibited an increased number of pro-inflammatory microglia, activation of the NOD-like receptor protein 3 (NLRP3) inflammasome, and enhanced IL-1β production in the TNC. Pharmacological inhibition of microglial activation reduced central sensitization in AR mice and was accompanied by remission of AR. Transcriptomics analysis revealed the inflammatory-prone characteristics of IL-4-treated microglia. IL-4 exposure enhanced lipopolysaccharide-stimulated NLRP3 inflammasome activation in microglia. Intracerebral injection of IL-4 neutralizing antibodies ameliorates neuroinflammation and central sensitization in AR mice and was accompanied by remission of AR. **Conclusions:** This research uncovers a previously unidentified mechanism wherein microglial neuroinflammation-induced central sensitization in the TNC contributes to AR, providing a new promising approach for the treatment of AR. IL-4 induces noncanonical pro-inflammatory-prone microglia and participates in microglial NLRP3 inflammasome activation in AR.

## Introduction

Allergic rhinitis (AR) is among the most widespread chronic inflammatory disorders of the respiratory tract, estimated to affect nearly 500 million people worldwide [1]. Currently, the mainstream academic view holds that AR is an immune dysfunction disorder, marked by the overactivation of the type 2 immune response [2]. Over recent years, substantial strides have been made in unraveling the complex networks leading to type 2 inflammation in AR [3]. However, the complex pathophysiological manifestations and clinical features of AR cannot be fully explained by attributing it solely to immune dysfunction [4,5]. Therefore, further understanding of the pathophysiology of AR is required.

The finding that the nervous system is an important player in allergic disease development marked a paradigm shift in allergology [6–8]. Most major AR symptoms, including nasal itch, rhinorrhea, and sneezing, can be attributed to an up-regulation of neural activity [7]. Furthermore, emerging evidence demonstrates that cross talk between the immune and nervous systems within nasal mucosa is also crucial in driving AR pathogenesis [9]. Notably, some investigators have extended this paradigm shift beyond the peripheral nervous system. For instance, the central nervous system (CNS) is hypothesized to contribute to AR through the nose–brain axis [10]. While the regulatory role of the CNS in AR has been theoretically postulated, the precise mechanisms underlying this relationship remain elusive. Central sensitization, a phenomenon characterized by hyperresponsiveness of the CNS to harmful stimuli, results in pathological amplification of responses to normal or subthreshold afferent inputs and abnormal changes in neural output patterns [11]. An elegant study on a rhesus allergic asthma model implicated central sensitization in the pathophysiology of allergic diseases [12]. This study demonstrated that allergen exposure increases the excitability of second-order lung afferent neurons in the nucleus tractus solitarius [12]. Given the pathophysiological similarities between AR and asthma, it is reasonable to speculate that central sensitization may also be operative in AR. Neuroimaging evidence has provided initial insights into the aberrant neural plasticity in AR. Using functional magnetic resonance imaging (fMRI), Callebaut et al. [13] found that individuals with AR showed significantly stronger activation in many brain regions after nasal histamine provocation compared to normal controls. Our team’s resting-state fMRI research demonstrated that AR patients exhibited heightened anterior cingulate cortex activity, which was positively correlated with the visual analog scale symptom score [14].

An important feature of central sensitization is the increased synaptic efficacy and excitability of secondary neurons within the CNS [11,15]. Neural tracing studies in cats and rats revealed that the secondary neurons of the afferent neural pathway projecting from nasal regions are mainly situated in the trigeminal nucleus caudalis (TNC) of the brain stem [16,17]. This anatomical evidence supports the critical role of the TNC in maintaining the functional homeostasis of the nose. Moreover, the TNC is recognized as the primary hub for integrating and processing information from harmful craniofacial stimuli [18]. Therefore, we hypothesize that the TNC region, serving as the primary central relay station of the nasal nervous system, represents one of the key regions implicated in central sensitization associated with AR. Further studies on the mechanisms of central sensitization uncovered that the phenomenon did not depend on sustained peripheral neuronal activity [15]. Instead, microglia-mediated neuroinflammation plays a critical role in both the development and maintenance of central sensitization [19,20]. Evidence of microglial neuroinflammation has been identified in animal models of allergic diseases such as atopic dermatitis, asthma, and AR [21–23]. However, the molecular mechanisms underlying microglial neuroinflammation in AR and whether this mediates central sensitization, thereby contributing to AR pathophysiology, are not yet fully clarified.

This research focuses on identifying the occurrence of central sensitization in AR and understanding how central sensitization impacts AR pathophysiology. Furthermore, we aim to investigate the underlying mechanisms involving microglia–neuron interactions in AR-associated central sensitization and explore the related immune–brain axis in AR.

## Results

### OVA-induced AR led to central sensitization in the TNC of mice

**Fig. 1.**
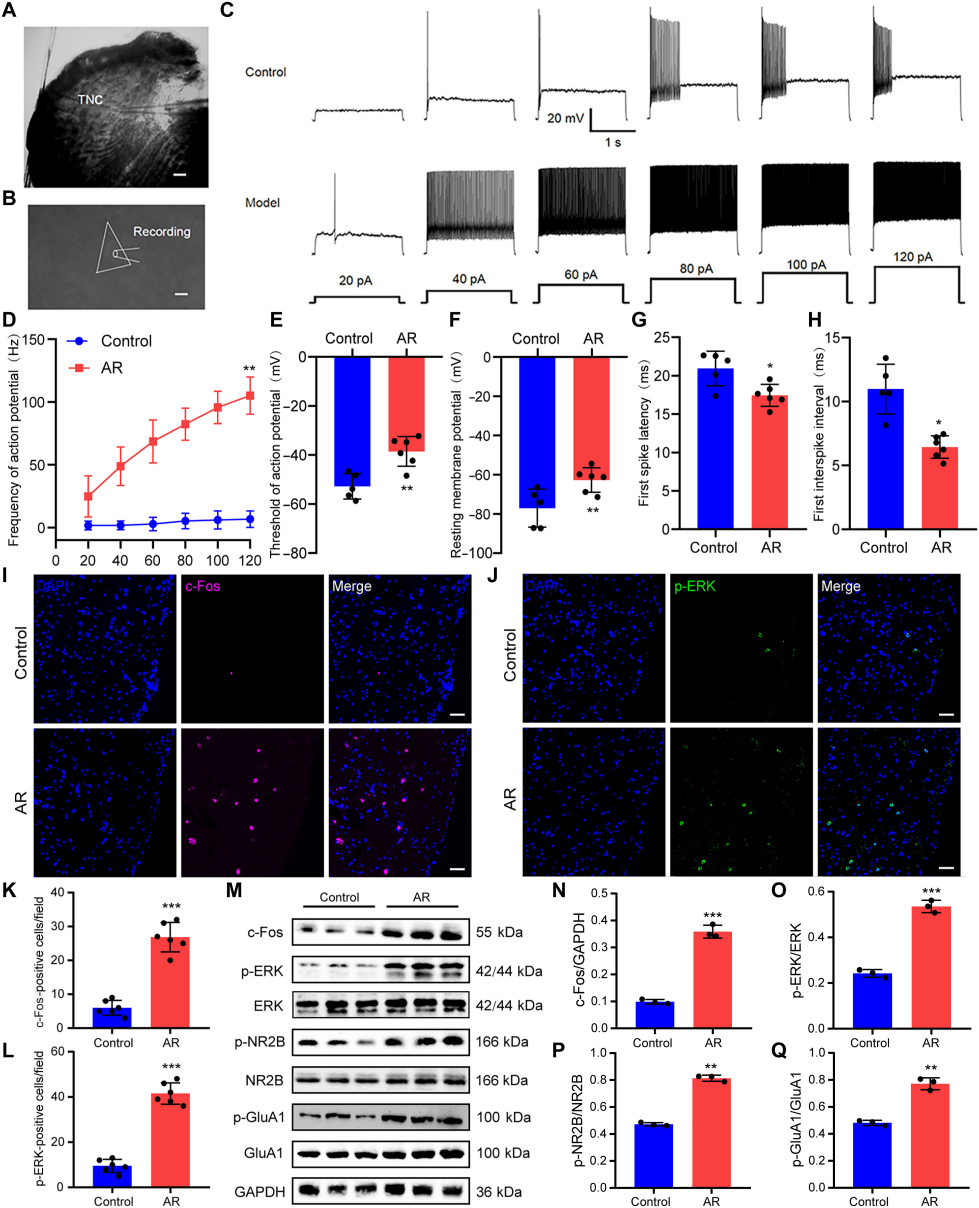
Allergic rhinitis (AR) led to central sensitization in the trigeminal nucleus caudalis (TNC). (A) Representative image showing a TNC slice with a recording pipette. Scale bar, 50 μm. (B) Representative image showing the whole-cell formation in a TNC neuron. Scale bar, 10 μm. (C) Responses to current injections in TNC neurons. (D) Frequency of action potentials evoked by depolarizing current injections in TNC neurons. *N* = 5 to 6 mice per group. (E) The action potential threshold of TNC neurons. *N* = 5 to 6 mice per group. (F) The resting membrane potential of TNC neurons. *N* = 5 to 6 mice per group. (G) The first spike latency of TNC neurons. *N* = 5 to 6 mice per group. (H) The first interspike interval (ISI) of action potentials in TNC neurons. *N* = 5 to 6 mice per group. (I and J) Representative images of immunofluorescence staining with c-Fos and p-ERK. Scale bars, 50 μm. (K and L) Number of c-Fos-positive cells and number of p-ERK-positive cells. *N* = 3 mice per group. (M) Western blotting for the detection of c-Fos, p-ERK, p-NR2B, NR2B, p-GluA1, and GluA1 in the TNC. (N to Q) Quantification analysis of the protein expression of c-Fos, p-ERK, p-NR2B, and p-GluA1. *N* = 3 mice per group. *P < 0.05; **P < 0.01; ***P < 0.001.

We used whole-cell patch-clamp recordings to examine phenotypic changes in the electrophysiological properties of TNC neurons from mouse brain slices (Fig. 1A and B). Depolarizing current injections evoked action potentials (APs) of a higher frequency in TNC neurons from AR mice than in those from control mice (Fig. 1C and D). Compared with control mice, AR mice showed significantly decreased AP thresholds, depolarized resting membrane potentials, reduced first spike latencies, and shortened first interspike intervals in TNC neurons (Fig. 1E to H).

Next, we examined changes in biochemical markers in the TNC. c-Fos and p-ERK are established markers for central sensitization and neuronal activation [24]. The TNC of AR mice contained more c-Fos^+^ and p-ERK^+^ cells than that of control mice, as evidenced by immunofluorescence (Fig. 1I to L). In addition, Western blotting showed that ovalbumin (OVA) stimulation significantly up-regulated c-Fos and p-ERK at the protein level in the TNC of mice (Fig. 1M to Q). Glutamate receptors, including *N*-methyl-d-aspartate receptor (NMDAR) and α-amino-3-hydroxy-5-methyl-4-isoxazolepropionic acid receptor (AMPAR), are critical components of excitatory neurotransmission in the CNS. Phosphorylation of the NMDAR subunit NR2B and the AMPAR subunit GluA1 is essential for synaptic plasticity and mediates central sensitization under pathological conditions [11,25]. AR caused a marked rise in the phosphorylation of NR2B and GluA1 within the TNC of mice (Fig. 1M to Q).

### Chemogenetic inhibition of the TNC neurons relieved OVA-induced AR

**Fig. 2.**
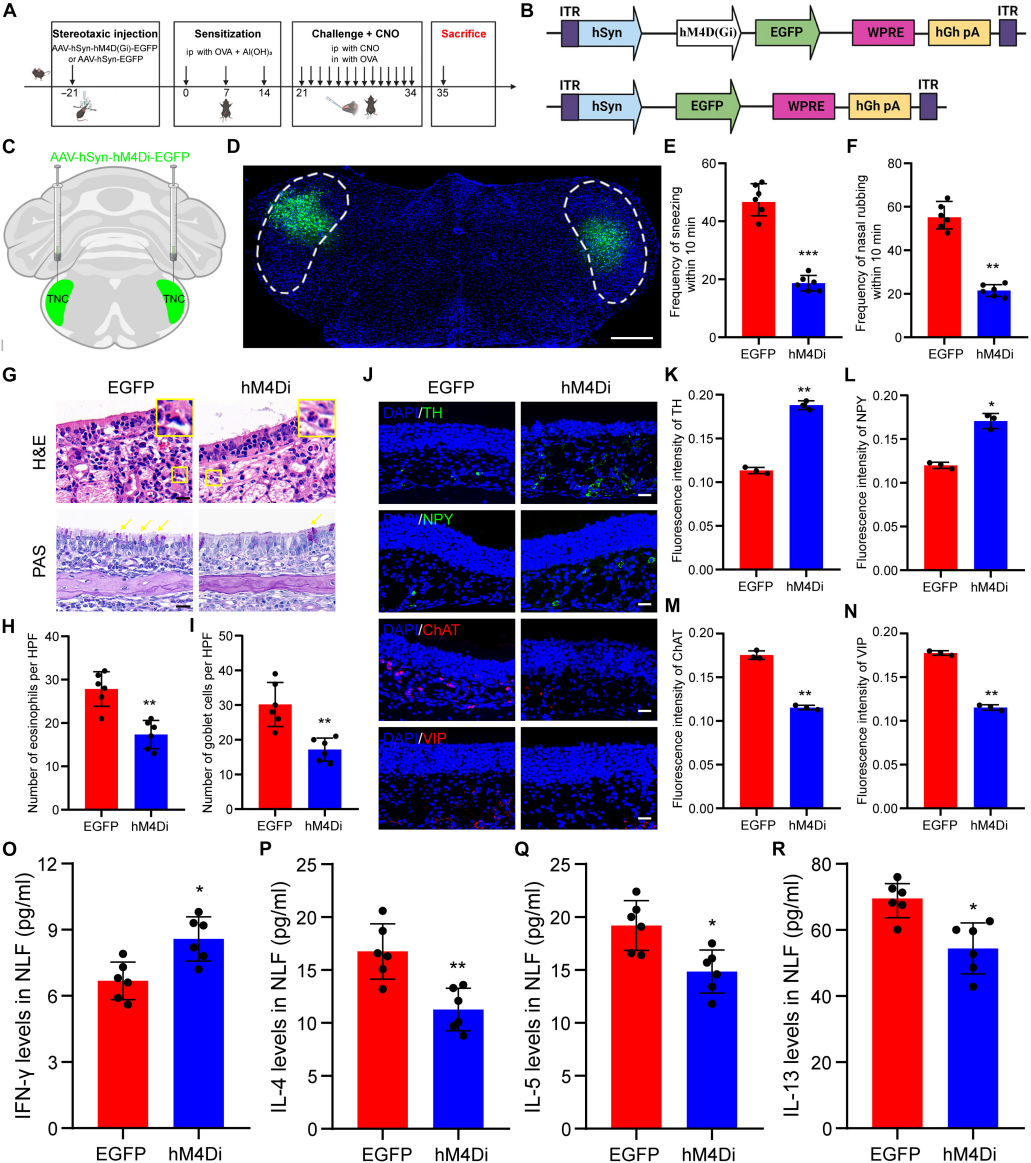
Chemogenetic inhibition of the TNC neurons improved AR. (A) Flowchart of experimental design and effects of the chemogenetic inhibition of the TNC neurons of AR mice. (B) Illustration of the AAV-hSyn-hM4D(Gi)-EGFP/AAV-hSyn-hM4Di-EGFP construct. (C) Schematic diagram showing chemogenetic virus injection into the TNC of mice. (D) hM4Di-EGFP fluorescence localization in the TNC region. Scale bar, 500 μm. (E and F) Quantification of nasal rubbing and sneezing episodes within 10 min. *N* = 6 mice per group. (G) Hematoxylin–eosin (H&E) and periodic acid–Schiff (PAS) staining of nose sections. Scale bars, 20 μm. (H and I) Quantification of eosinophil and goblet cell counts in a ×400 high-power field. *N* = 6 mice per group. (J) Immunofluorescence staining with sympathetic nerve markers (tyrosine hydroxylase [TH] and neuropeptide Y [NPY]) and parasympathetic nerve markers (choline acetyltransferase [ChAT] and vasoactive intestinal peptide [VIP]) in the nasal mucosa. Scale bars, 20 μm. (K to N) Quantification of the immunofluorescence intensity of TH, NPY, ChAT, and VIP in the nasal mucosa. *N* = 3 mice per group. (O to R) Contents of interferon-γ (IFN-γ), interleukin (IL)-4, IL-5, and IL-13 in the nasal lavage fluid (NLF). *N* = 6 mice per group. *P < 0.05; **P < 0.01; ***P < 0.001. ip, intraperitoneal; OVA, ovalbumin; CNO, clozapine-*N*-oxide; in, intranasal; HPF, high-power field.

To test the hypothesis that activation of TNC neurons can contribute to AR, we developed a mouse model of OVA-induced AR, and performed chemogenetic inhibition of TNC neurons by adeno-associated viruses (AAVs) carrying hM4D(Gi)-EGFP (Fig. 2A and B). We assessed EGFP fluorescence in the TNC post hoc to confirm the accurate location of the virus injections (Fig. 2C and D). As expected, the chemogenetic suppression of TNC neuronal activity resulted in a significant decrease in the nose scratching and sneezing frequency of mice compared with that of AR mice with AAV-hSyn-EGFP (control group) (Fig. 2E and F). Moreover, the AAV-hSyn-hM4D(Gi)-EGFP-treated mice exhibited reduced eosinophilic infiltration and attenuated goblet cell hyperplasia in their nasal mucosa (Fig. 2G to I). Compared to those of the control group, immunofluorescence results demonstrated significantly elevated levels of the sympathetic nerve markers neuropeptide Y (NPY) and tyrosine hydroxylase (TH), and reduced levels of the cholinergic nerve markers vasoactive intestinal peptide (VIP) and choline acetyltransferase (ChAT), in the AAV-hSyn-hM4D(Gi)-EGFP-treated group (Fig. 2J to N). The AAV-hSyn-hM4D(Gi)-EGFP-treated group showed significantly decreased levels of Th2 cytokines (interleukin [IL]-4, IL-5, and IL-13), and a notable elevation in the Th1 cytokine interferon-γ (IFN-γ) compared to the control group (Fig. 2O to R).

### Inhibition of microglial activation attenuated central sensitization in the TNC of AR mice

**Fig. 3.**
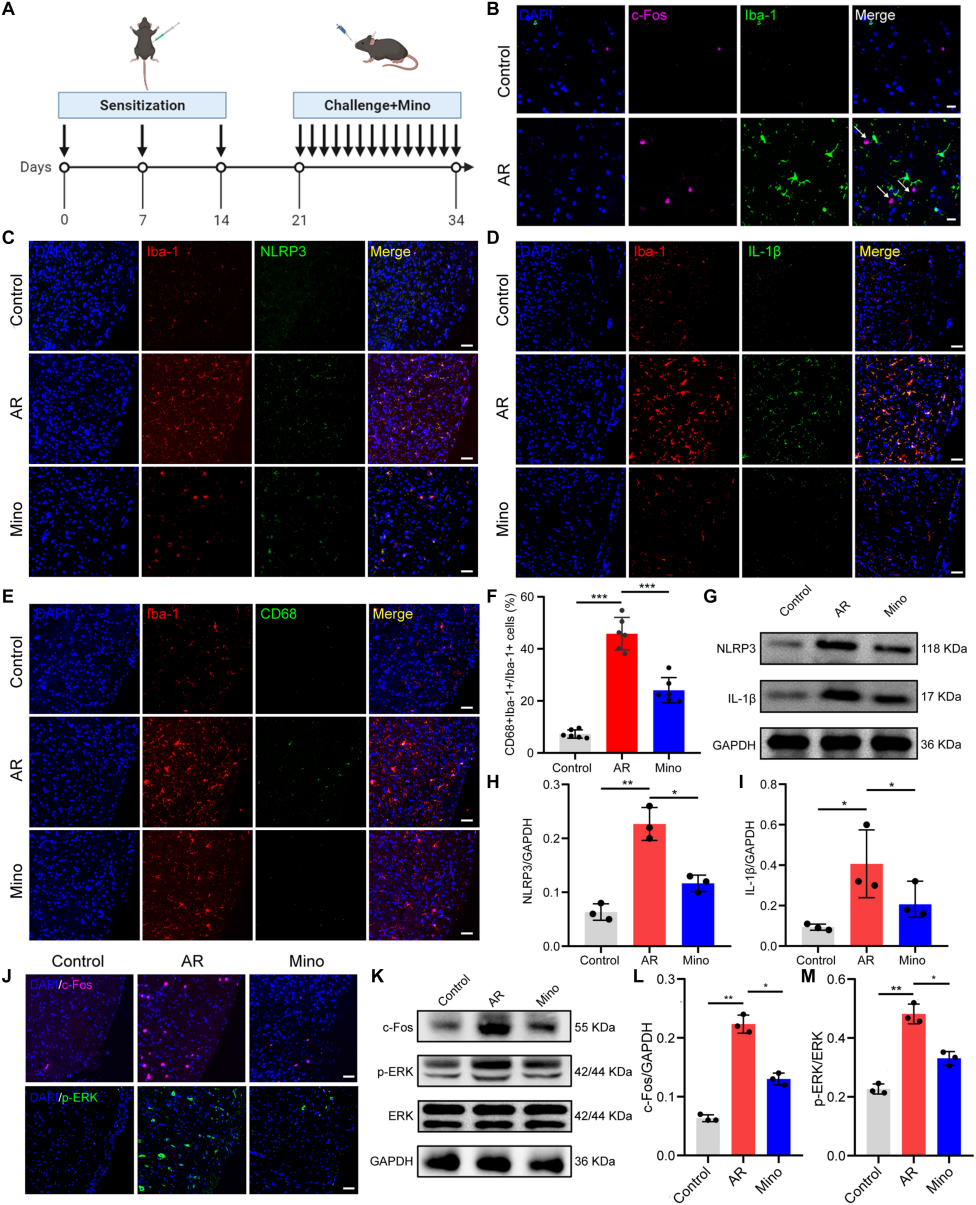
Inhibition of microglia activation attenuated NOD-like receptor protein 3 (NLRP3) signaling and central sensitization in AR. (A) Schematic diagram of the experimental design. (B) Double immunofluorescence staining for c-Fos and Iba-1. Scale bars, 20 μm. (C) Double immunofluorescence staining for Iba-1 and NLRP3. Scale bars, 50 μm. (D) Double immunofluorescence staining for Iba-1 and IL-1β. Scale bars, 50 μm. (E) Double immunofluorescence staining for Iba-1 and CD68. (F) Quantification of the percentage of CD68-positive microglia. *N* = 6 mice per group. (G) Western blotting for the detection of NLRP3 and IL-1β. (H and I) Quantification analysis of the protein expression of NLRP3 and IL-1β. *N* = 3 mice per group. (J) Immunofluorescence staining with c-Fos and p-ERK in the TNC. Scale bars, 50 μm. (K) Western blotting for the detection of c-Fos and p-ERK of TNC tissues. (L and M) Quantification analysis of c-Fos and p-ERK protein expression. *N* = 3 mice per group. *P < 0.05; **P < 0.01; ***P < 0.001. Mino, minocycline.

NOD-like receptor protein 3 (NLRP3) inflammasome-mediated microglial inflammation is an important mechanism of central sensitization [24,26]. Therefore, we hypothesized that NLRP3 signaling may be involved in central sensitization in AR mice. An outline of the experimental procedure is provided in Fig. 3A. Immunostaining results indicated that the c-Fos^+^ cells were in close proximity to microglia (Iba-1^+^ cells) in the TNC of AR mice (Fig. 3B). Compared with control mice, double immunostaining showed a significant up-regulation of NLRP3 and downstream IL-1β expression in microglia within the TNC of AR mice (Fig. 3C and D). We also assessed the microglial marker Iba-1 and the microglia activation marker CD68 (Fig. 3E). In comparison with control mice, AR mice had a notable elevation in CD68^+^ microglia within the TNC, while the microglia inhibitor minocycline reversed this trend (Fig. 3F). The proportion of M1 pro-inflammatory microglia within the TNC was increased in AR mice (Fig. S1). Moreover, OVA administration markedly increased the NLRP3 and IL-1β expression in the TNC of AR mice compared with that of control mice, but daily minocycline treatment remarkably decreased NLRP3 and IL-1β expression (Fig. 3G to I). More importantly, minocycline administration resulted in a marked reduction of c-Fos^+^ and p-ERK^+^ cells within the TNC compared to AR mice (Fig. 3J). The expression level of c-Fos and p-ERK in the TNC tissues of AR mice was also decreased after minocycline administration (Fig. 3K to M).

### Inhibition of microglial activation relieved OVA-induced AR

**Fig. 4.**
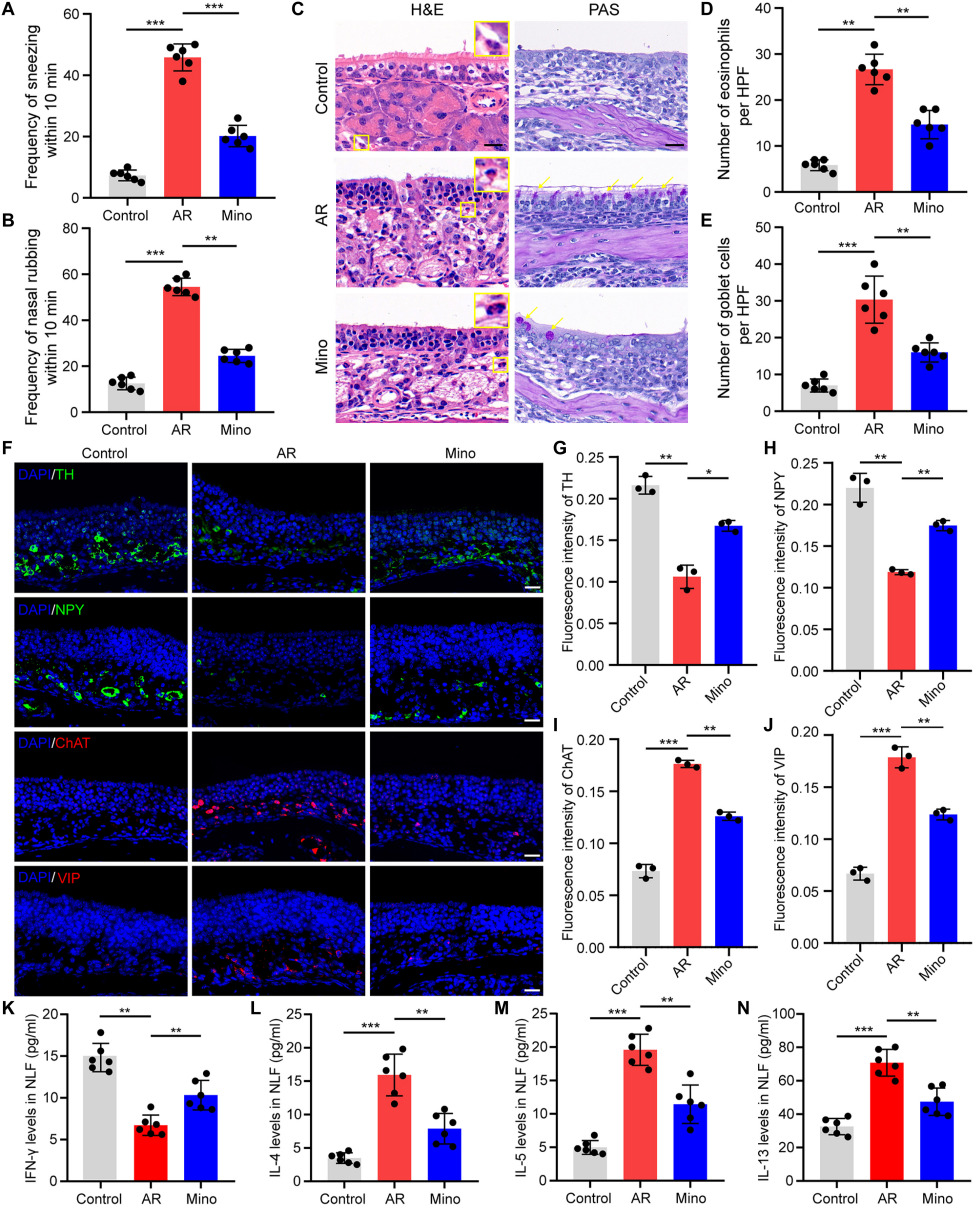
Inhibition of microglia activation improved AR. (A and B) Quantification of nasal rubbing and sneezing episodes within 10 min. *N* = 6 mice per group. (C) H&E and PAS staining of nose sections. Scale bars, 20 μm. (D and E) Quantification of eosinophil and goblet cell counts in a ×400 high-power field. *N* = 6 mice per group. (F) Immunofluorescence staining with sympathetic nerve markers (TH and NPY) and parasympathetic nerve markers (ChAT and VIP) in the nasal mucosa. Scale bars, 20 μm. (G to J) Quantification of the immunofluorescence intensity of TH, NPY, ChAT, and VIP. *N* = 3 mice per group. (K to N) Content of IFN-γ, IL-4, IL-5, and IL-13 in the NLF. *N* = 6 mice per group. *P < 0.05; **P < 0.01; ***P < 0.001.

Minocycline administration caused a significant reduction in sneezing and nasal rubbing in AR mice (Fig. 4A and B). In addition, it significantly mitigated goblet cell hyperplasia and eosinophil infiltration in the nasal mucosa of AR mice (Fig. 4C to E). Immunofluorescence staining revealed that autonomic dysfunction in AR mice was ameliorated by minocycline, as evidenced by the up-regulation of TH and NPY and down-regulation of ChAT and VIP in the nasal mucosa (Fig. 4F to J). Minocycline treatment also modulated Th1/Th2 cytokine production. There was a marked decrease in IL-4, IL-5, and IL-13 levels and an elevation in IFN-γ levels in the nasal lavage fluid (NLF) of AR mice (Fig. 4K to N).

### Blockade of NLRP3 signaling attenuated pro-inflammatory microglia-mediated neuronal activation

**Fig. 5.**
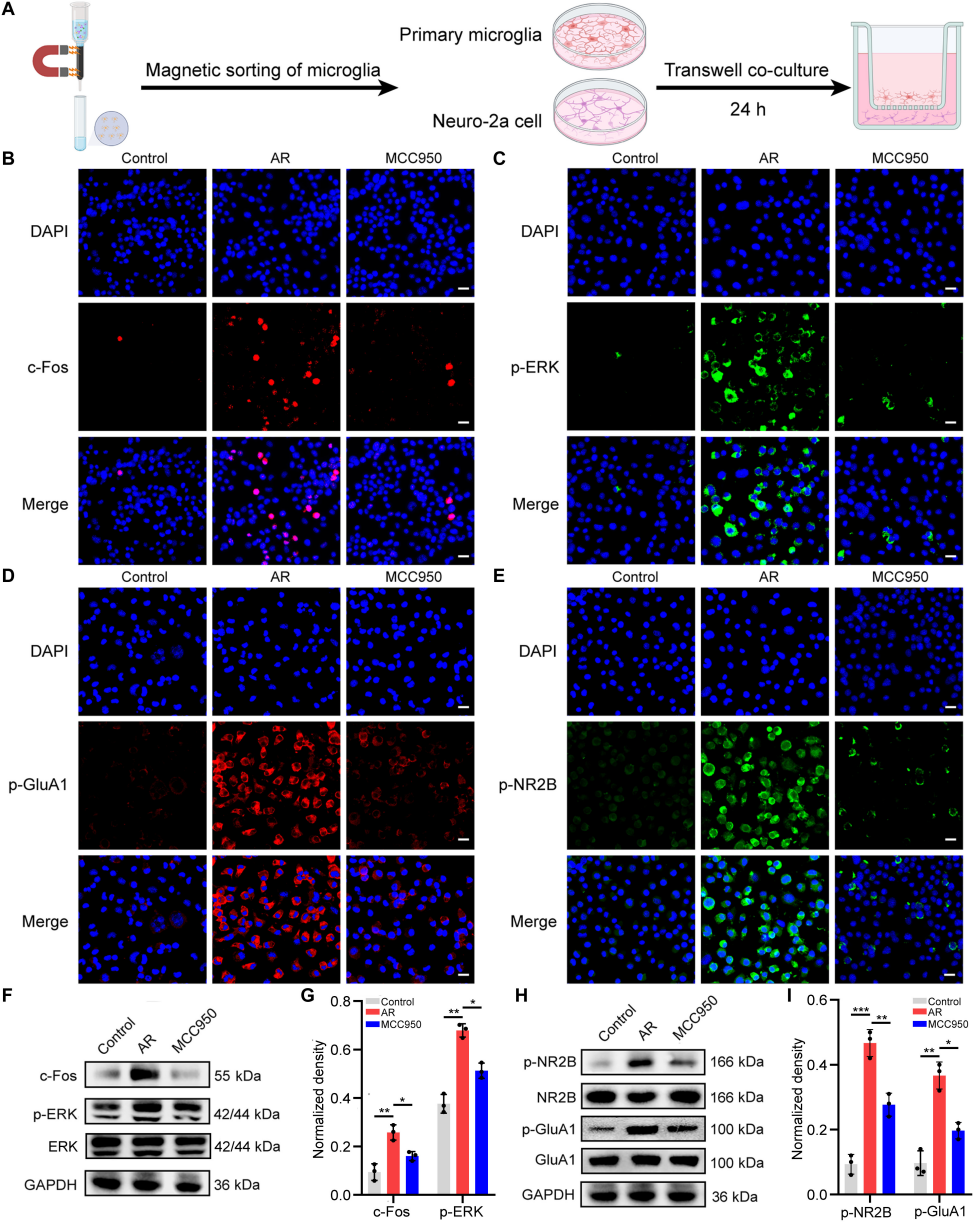
Blockade of NLRP3 signaling attenuated AR-derived microglia-mediated neuronal activation. (A) Experimental design schematic of microglia–neuron co-culture. (B and C) Immunofluorescent staining of c-Fos and p-ERK in Neuro-2a cells. Scale bars, 20 μm. (D and E) Immunofluorescent staining of p-NR2B and p-GluA1 in Neuro-2a cells. Scale bars, 20 μm. (F) Western blotting for the detection of c-Fos and p-ERK expression in Neuro-2a cells. (G) Quantification analysis of c-Fos and p-ERK protein expression. (H) Western blotting for the detection of NR2B, GluA1, p-NR2B, and p-GluA1 expression in Neuro-2a cells. (I) Quantification analysis of NR2B and GluA1 phosphorylation levels. *P < 0.05; **P < 0.01; ***P < 0.001.

We verified that microglia modulate neuronal activation using a microglia–neuron co-culture system (Fig. 5A). Immunofluorescence staining showed that c-Fos and p-ERK, markers of neuronal activation, were up-regulated in Neuro-2a cells co-cultured with primary microglia isolated from AR mice, whereas MCC950 treatment effectively reversed this expression trend (Fig. 5B and C). In addition, the phosphorylation levels of NR2B and GluA1, markers of synaptic plasticity, were high in Neuro-2a cells in the co-culture system, whereas MCC950 treatment effectively reversed this change (Fig. 5D and E). Western blotting corroborated these immunofluorescence staining results (Fig. 5F to I). Additionally, the downstream effector of the NLRP3 inflammasome, IL-1β, was neutralized in mice to further elucidate its contribution to AR-related central sensitization. IL-1β neutralizing antibody (NAb) treatment decreased neuronal activation in the TNC and alleviated sneezing and nose-scratching behaviors in AR mice (Fig. S2).

### AR-induced BBB changes increased peripheral IL-4 access to the TNC

**Fig. 6.**
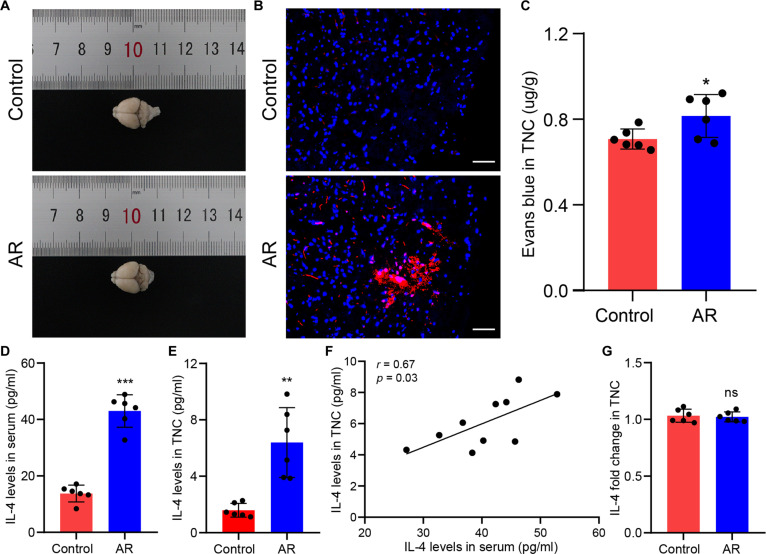
AR-induced blood–brain barrier (BBB) changes facilitated peripheral IL-4 entry into the TNC. (A) Bright-field image of Evans blue (EB) permeation in mice. (B) Confocal microscopy image of TNC slices. Scale bars, 50 μm. (C) Content of EB in the TNC of mice. *N* = 6 mice per group. (D) IL-4 levels in the serum. *N* = 6 mice per group. (E) IL-4 Levels in the TNC. *N* = 6 mice per group. (F) Correlation analysis between serum and TNC IL-4 levels. (G) Expression levels of the IL-4 gene in the TNC. *P < 0.05; **P < 0.01; ***P < 0.001.

Given the significance of the blood–brain barrier (BBB) in CNS homeostasis, BBB permeability was evaluated with Evans blue (EB) dye. EB extravasation was detected in the TNC of AR mice compared to controls (Fig. 6A and B). Relative quantitative data also showed a notable rise in EB content within the TNC of AR mice (Fig. 6C). The results suggested that BBB leakage was present within the TNC of AR mice. There is substantial evidence that peripheral inflammatory factors can enter the brain through a weakened BBB, thereby triggering neuroinflammation [27,28]. Thus, we asked whether IL-4, a central mediator of allergic inflammation, could cross the BBB in AR mice. There was a marked rise in IL-4 levels in the peripheral circulation and TNC tissues of AR mice in comparison with those in control mice (Fig. 6D and E). A positive correlation was observed between IL-4 levels in the peripheral circulation of AR mice and IL-4 levels in the TNC (Fig. 6F). Furthermore, no significant difference in IL-4 messenger RNA (mRNA) abundance was detected between controls and AR mice through quantitative real-time polymerase chain reaction (qRT-PCR) assays, as represented in Fig. 6G. Beyond IL-4, we measured the levels of other type 2 cytokines, IL-5 and IL-13, in the TNC. The results showed that compared to those in the control group, IL-13 levels were significantly increased in the TNC of AR mice, while IL-5 levels showed no significant difference (Fig. S3).

### Transcriptomics analysis revealed the inflammatory-prone feature of IL-4-treated microglia

**Fig. 7.**
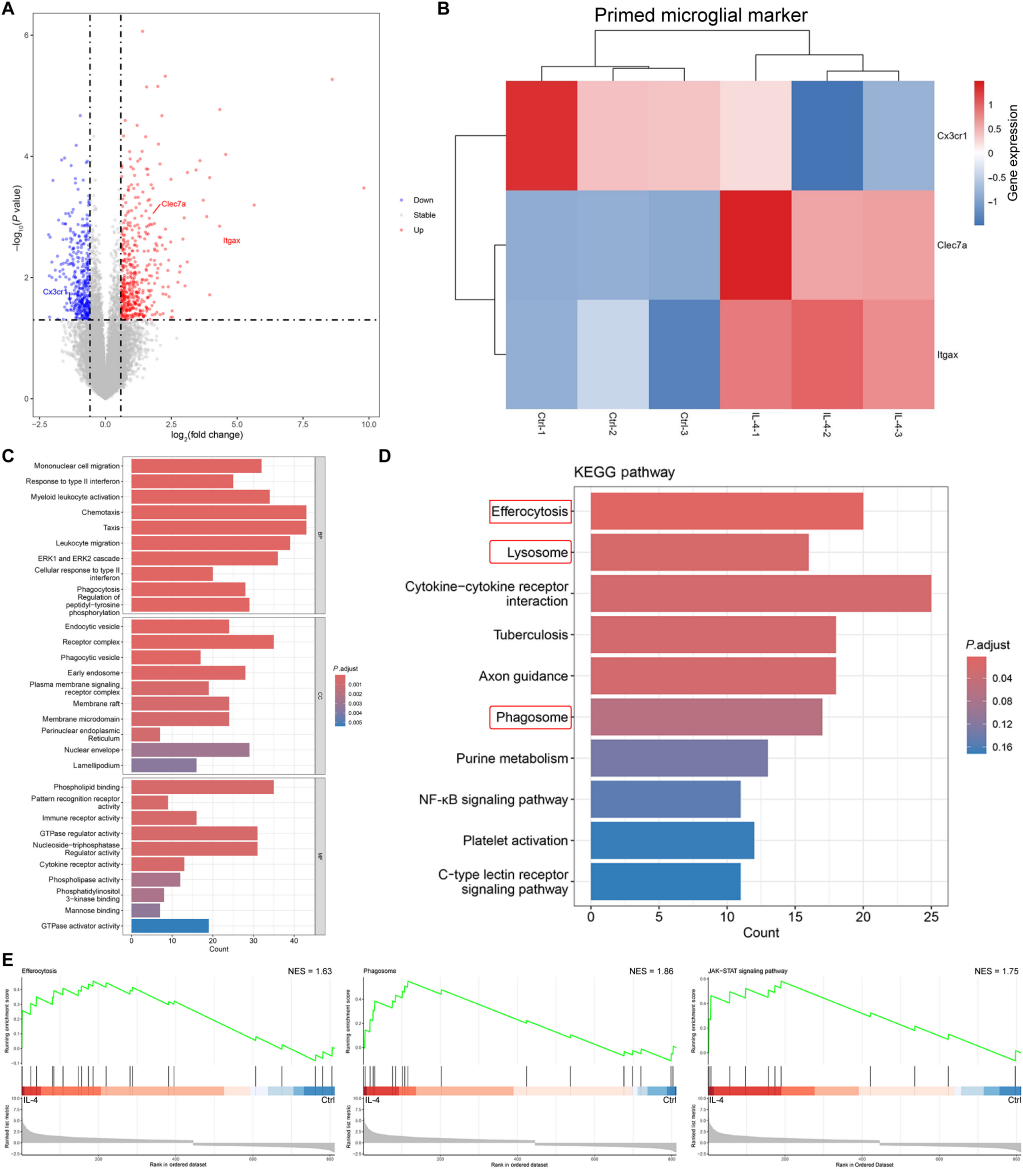
The transcriptomic profile of IL-4-treated microglia. (A) Volcano plot of differentially expressed genes (DEGs) between IL-4-treated and control primary mouse microglia. (B) Normalized expression levels of “microglia priming”-related gene in control and IL-4-treated microglia. (C) Gene Ontology (GO) enrichment analysis of DEGs between IL-4-treated and control microglia. (D) Top 10 enriched pathways in IL-4-treated microglia by Kyoto Encyclopedia of Genes and Genomes (KEGG). (E) Enrichment of the efferocytosis, phagosome, and JAK–STAT signaling pathways in IL-4-treated microglia shown by gene set enrichment analysis (GSEA). *P < 0.05; **P < 0.01; ***P < 0.001. BP, Biological Process; CC, Cellular Component; MF, Molecular Function; NES, normalized enrichment score.

We explored the impact of IL-4 on microglia through a reanalysis of the GSE157891 dataset from the Gene Expression Omnibus database, seeking to obtain a panorama of the transcriptomic profile of IL-4-induced primary mouse microglia. There were 838 differentially expressed genes (DEGs) between the control and IL-4 treatment groups. Within the DEGs, Clec7a and Itgax were up-regulated, while Cx3cr1 was down-regulated (Fig. 7A and B). In addition, the DEGs were enriched in the “lysosome” and “phagosome” pathways, as shown by Gene Ontology (GO) and Kyoto Encyclopedia of Genes and Genomes (KEGG) pathway analysis (Fig. 7C and D). Additionally, the gene set enrichment analysis revealed that the “phagosome” pathway was significantly enriched in IL-4-treated microglia (Fig. 7E). These gene and pathway changes in IL-4-treated microglia were consistent with the characteristics of “microglia priming” [29]. Furthermore, the primed microglia are more sensitive to secondary inflammatory signals, which in turn elicit an exaggerated inflammation [29]. The DEG functions were enriched in the “efferocytosis” pathway.

**Fig. 8.**
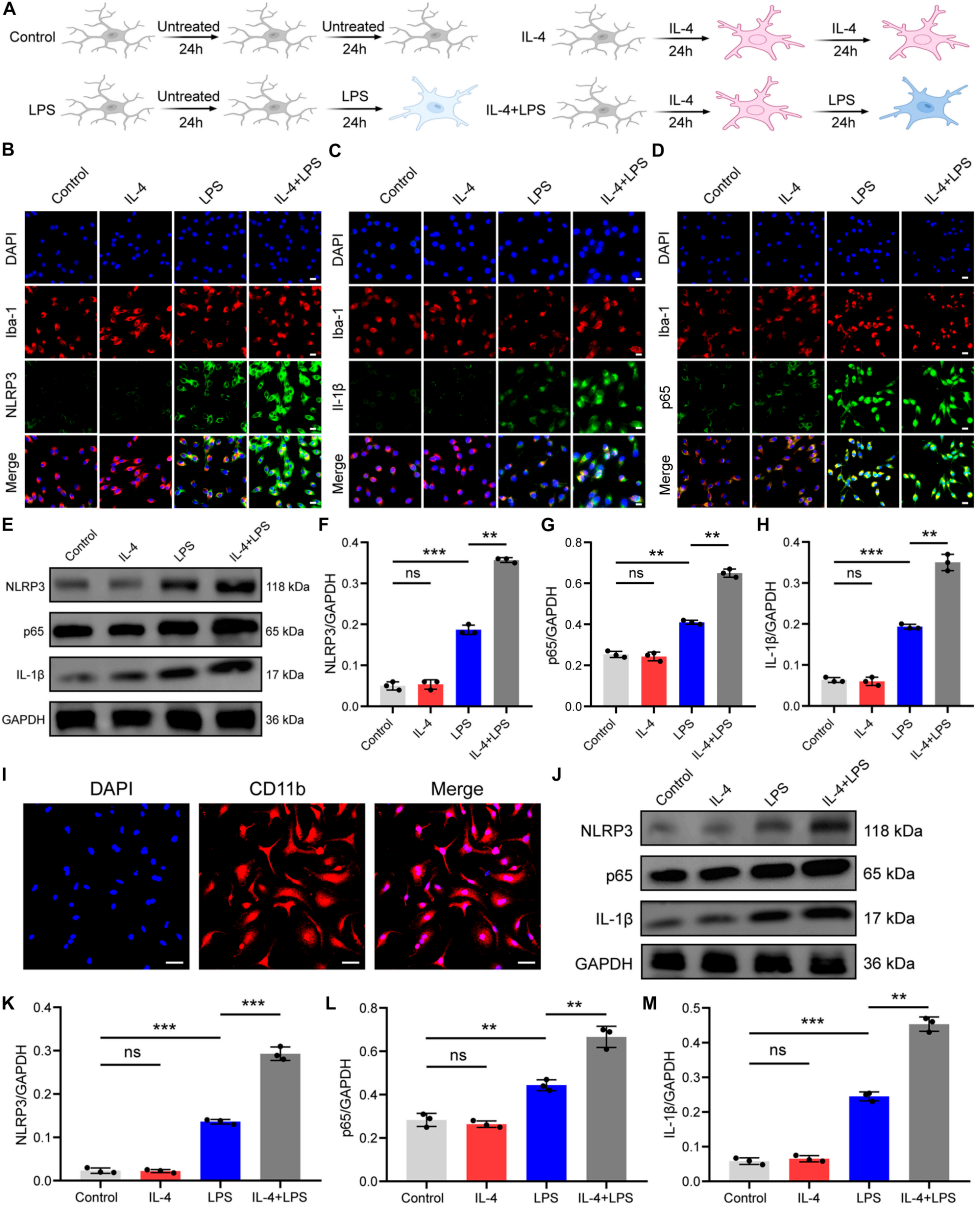
IL-4 exposure enhanced lipopolysaccharide (LPS)-induced NLRP3 signaling in microglia. (A) Flowchart of experimental design. (B) Double immunofluorescence staining for Iba-1 and NLRP3 in BV-2 cells. Scale bars, 20 μm. (C) Double immunofluorescence staining for Iba-1 and IL-1β in BV-2 cells. Scale bars, 20 μm. (D) Double immunofluorescence staining for Iba-1 and p65 in BV-2 cells. Scale bars, 20 μm. (E) Western blotting analysis of NLRP3, IL-1β, and p65 expression in BV-2 cells. (F to H) Quantification analysis of NLRP3, IL-1β, and p65 expression. (I) Immunofluorescent staining of CD11b in primary mouse microglia. (J) Western blotting analysis of NLRP3, IL-1β, and p65 expression in primary mouse microglia. (K to M) Quantification analysis of NLRP3, IL-1β, and p65 expression. *P < 0.05; **P < 0.01; ***P < 0.001.

### IL-4 exposure enhanced LPS-induced NLRP3 signaling in microglia

To further explore the potential pro-inflammatory effects of IL-4 in microglia, BV-2 cells and primary mouse microglia were pretreated with IL-4 for 24 h and subsequently exposed to lipopolysaccharide (LPS) for another 24 h while maintaining IL-4 presence (Fig. 8A). The results suggest that IL-4 priming augmented LPS-stimulated NLRP3 inflammasome activation in BV-2 cells, with increased NLRP3 and IL-1β expression and enhanced expression and nuclear translocation of NF-κB p65 (Fig. 8B to H). IL-4 pretreatment was also found to augment the secretion of M1-associated cytokines (IL-1β and IL-6) and inhibit the expression of the M2-associated cytokine IL-10 in microglia following LPS stimulation (Fig. S4). In addition, we also validated the noncanonical pro-inflammatory effect of IL-4 on primary microglia (Fig. 8I). The results showed that IL-4 pretreatment further increased the LPS-induced expression of NLRP3, IL-1β, and NF-κB p65 in primary microglia (Fig. 8J to M).

### Blocking IL-4 in the TNC attenuated the NLRP3 signaling and central sensitization of AR mice

**Fig. 9.**
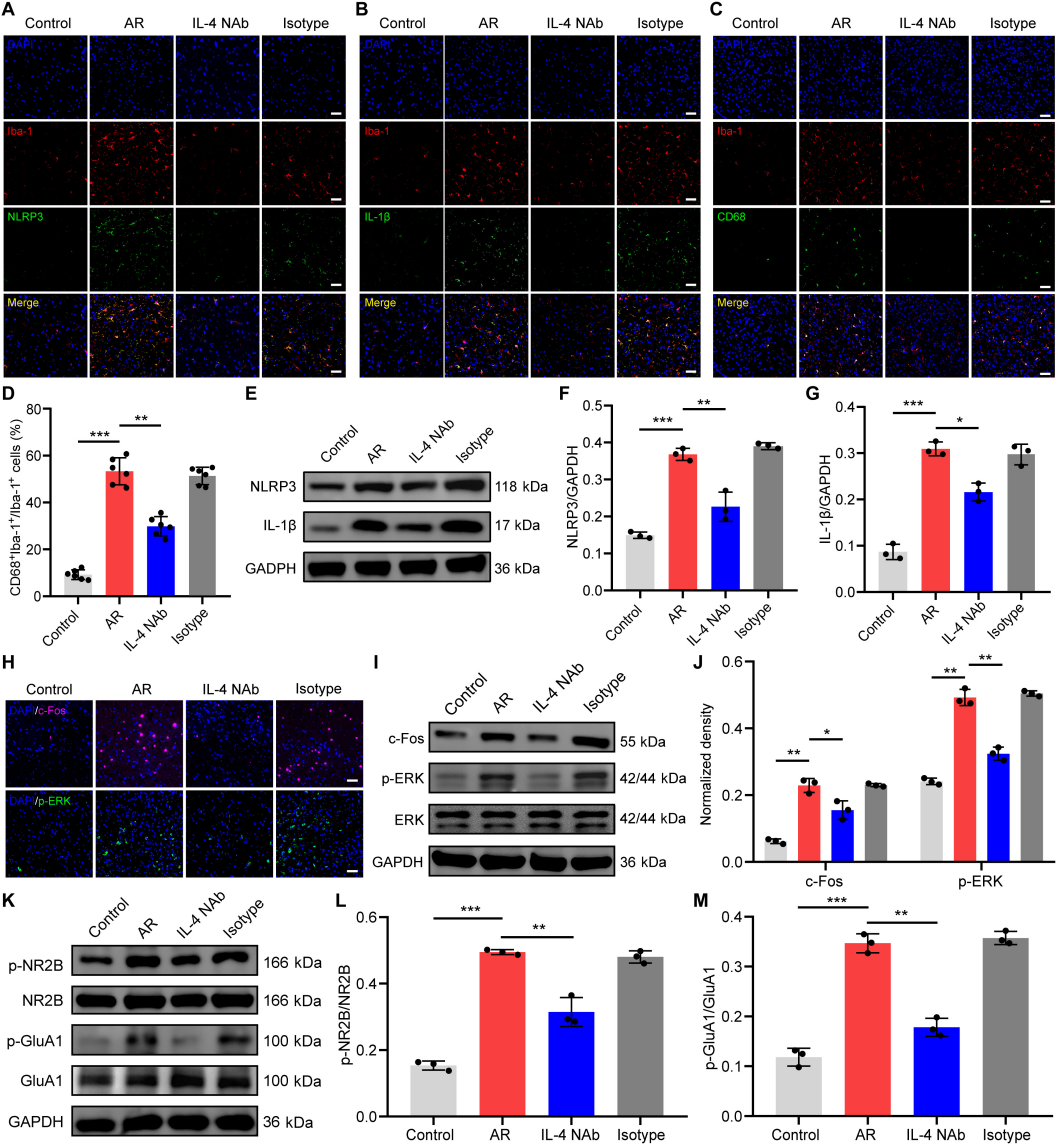
Intracerebral injection of IL-4 neutralizing antibodies ameliorates NLRP3 signaling and central sensitization in AR. (A) Double immunofluorescence staining for Iba-1 and NLRP3. Scale bars, 50 μm. (B) Double immunofluorescence staining for Iba-1 and IL-1β. Scale bars, 50 μm. (C) Double immunofluorescence staining for Iba-1 and CD68. Scale bars, 50 μm. (D) Quantitative analysis of the percentage of CD68-positive microglia. *N* = 6 mice per group. (E) Western blotting analysis of NLRP3 and IL-1β. (F and G) Quantification analysis of the protein expression of NLRP3 and IL-1β. *N* = 3 mice per group. (H) Immunofluorescence staining for c-Fos and p-ERK. (I) Western blotting analysis of the c-Fos and p-ERK of TNC tissues. (J) Quantification analysis of c-Fos and p-ERK protein expression. *N* = 3 mice per group. (K) Western blotting analysis of p-NR2B, NR2B, p-GluA1, and GluA1 in the TNC. (L and M) Quantification analysis of the protein expression of p-NR2B and p-GluA1. *N* = 3 mice per group. *P < 0.05; **P < 0.01; ***P < 0.001. NAb, neutralizing antibody.

In view of our experimental results and prior research, we postulated that IL-4 may facilitate microglial NLRP3 inflammasome activation in the TNC of AR mice. First, we examined IL-4 receptor expression on microglia within the TNC of mice. Immunofluorescence results demonstrated that microglia expressed IL-4 receptor alpha (IL-4 Rα) in both the control and AR groups (Fig. S5). IL-4 NAb treatment significantly decreased NLRP3 and IL-1β expression in microglia and the proportion of CD68^+^ microglia in the TNC of AR mice (Fig. 9A to G). Moreover, IL-4 NAb administration significantly attenuated the number of c-Fos^+^ and p-ERK^+^ cells in the TNC of AR mice (Fig. 9H). In addition, IL-4 NAb caused a significant decline in c-Fos and p-ERK protein expression and NR2B and GluA1 phosphorylation within the TNC of AR mice (Fig. 9I to M).

### Blocking IL-4 in the TNC relieved OVA-induced AR

**Fig. 10.**
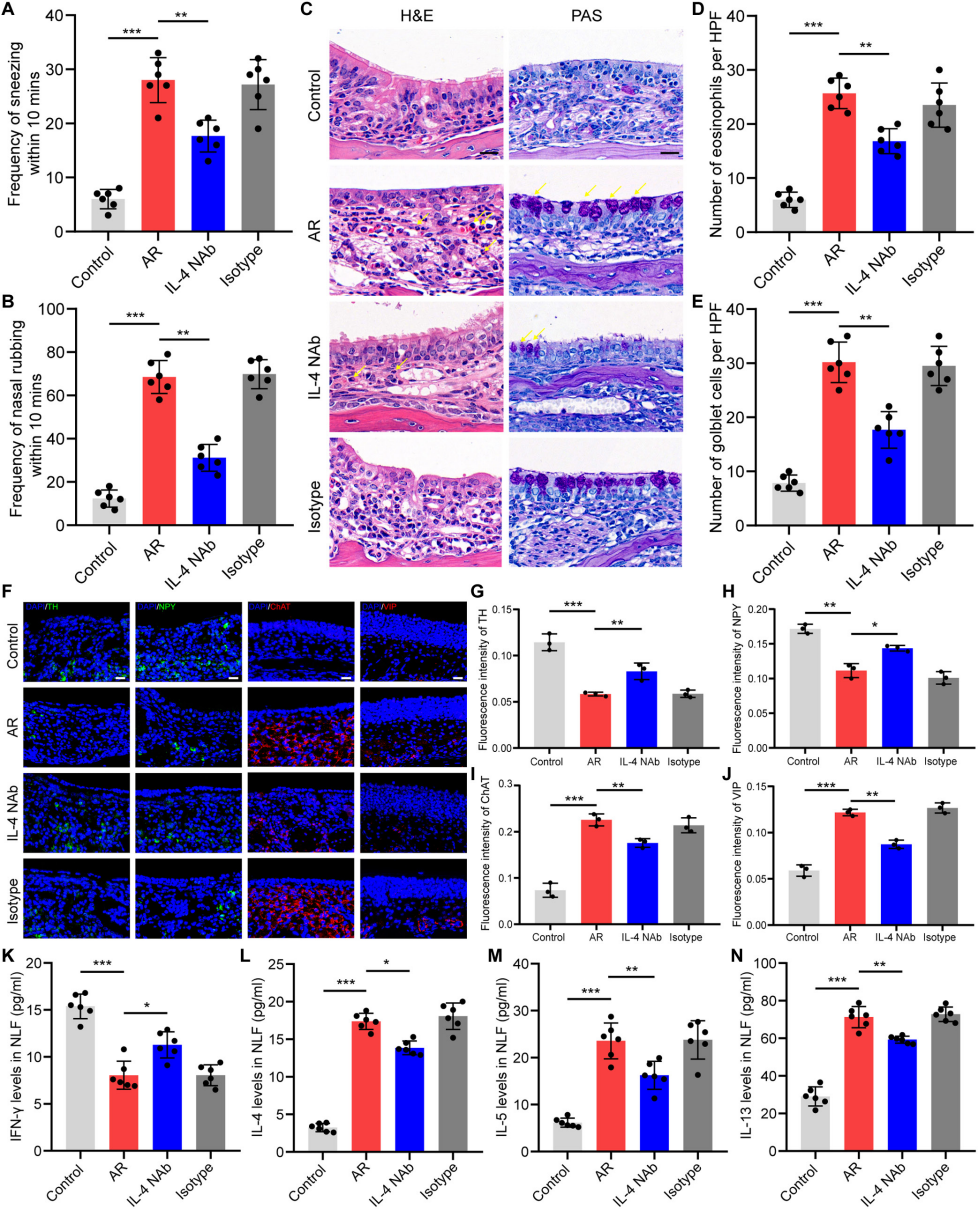
Intracerebral injection of IL-4 NAbs improved AR. (A and B) Quantification of nasal rubbing and sneezing episodes within 10 min. *N* = 6 mice per group. (C) H&E and PAS staining of nose sections. Scale bars, 20 μm. (D and E) Quantification of eosinophil and goblet cell counts in a ×400 high-power field. *N* = 6 mice per group. (F) Immunofluorescence staining with sympathetic nerve markers (TH and NPY) and parasympathetic nerve markers (ChAT and VIP). Scale bars, 20 μm. (G to J) Quantification of the immunofluorescence intensity of TH, NPY, ChAT, VIP. *N* = 3 mice per group. (K to N) Content of IFN-γ, IL-4, IL-5, and IL-13 in the NLF. *N* = 6 mice per group. *P < 0.05; **P < 0.01; ***P < 0.001.

IL-4 NAb treatment significantly diminished the occurrence of nose scratching and sneezing in AR mice (Fig. 10A and B). Furthermore, it markedly reduced goblet cell hyperplasia and eosinophil infiltration in the nasal mucosa of AR mice (Fig. 10C to E). Immunofluorescence staining revealed that autonomic dysfunction in AR mice was ameliorated by IL-4 NAb, as evidenced by the up-regulation of TH and NPY and down-regulation of ChAT and VIP in the nose (Fig. 10F to J). Moreover, IL-4 NAb treatment caused a marked decline in IL-4, IL-5, and IL-13 levels while elevating IFN-γ levels within the NLF of AR mice (Fig. 10K to N).

## Discussion

Central sensitization is a pivotal pathophysiological mechanism in many disorders associated with the somatosensory and visceral sensory systems, such as chronic pain, pruritus, and cough [11,30,31]. Central sensitization has also been widely mentioned in the literature as a potential CNS mechanism in allergic diseases [6–8]. However, the role of central sensitization in AR has not yet been definitively established. In this study, we first confirmed through neurotropic virus tracing that the second-order neurons of the nasal neural pathway of a mouse are primarily located in the TNC (Fig. S6), aligning with prior neural tracing research in rats and cats. Both our electrophysiological and biochemical results showed significant activation of TNC neurons in AR mice and provided direct evidence of central sensitization in AR. Remarkably, selective inhibition of neuronal activity in the TNC mitigated sneezing and nose scratching in AR mice. Furthermore, suppressing TNC neurons was found to ameliorate pathological alterations in the nasal mucosa of AR mice, including eosinophil infiltration and goblet cell hyperplasia. Overall, our study delivers the first demonstration of central sensitization in AR and further suggests that central sensitization in the TNC is an essential component in the pathophysiological mechanisms of AR.

Our study demonstrated that suppression of neuronal activity in the TNC improved both Th1/Th2 immune imbalance and autonomic dysfunction in the nose of AR mice. These findings demonstrate that central sensitization mediates autonomic dysfunction and immune imbalance in AR. The peripheral immune system and the brain are considered to communicate bidirectionally [32]. Specifically, the immune system and the CNS mainly communicate through the autonomic nervous system (ANS) and the neuroendocrine system [32]. A lot of research has proved the ANS is involved in the pathophysiology of AR [6,7]. Patients with AR exhibit parasympathetic hyperfunction and sympathetic hyperalgesia [33]. Hu et al. [34] found that inhibiting parasympathetic nerve activity attenuated group 2 innate lymphoid cell infiltration within the nasal mucosa of AR mice. In addition, cholinergic neurons have been reported to promote the polarization of Th2 cells in AR [35]. On an anatomical level, the TNC has synaptic connections with the superior salivatory nucleus (SSN), the high-level control center for parasympathetic nerves innervating nasal and lacrimal glands [36]. Our results revealed a significant increase in the number of activated neurons within the SSN of AR mice (Fig. S7). Chemogenetic inhibition of TNC neurons reduced SSN neuronal activation, providing preliminary evidence for a neural circuit basis underlying central sensitization in AR (Fig. S7). Furthermore, the TNC also projects to the hypothalamus, the higher-level center for the ANS and neuroendocrine system [18]. These neural connections may provide the basis for aberrant autonomic activity and peripheral immune dysfunction mediated by central sensitization in the TNC.

Microglial neuroinflammation is often considered one of the main causes of central sensitization [19,20]. This study revealed significant microglia activation and enhanced NLRP3 inflammasome signaling in the TNC of AR mice, while minocycline treatment effectively suppressed NLRP3 inflammasome activation in microglia. Furthermore, minocycline administration attenuated central sensitization within the TNC of AR mice. Administration of the NLRP3 inflammasome inhibitor MCC950 reduced the activation of neuronal cell lines by primary microglia isolated from AR mice. These observations point to microglial NLRP3 inflammasome activation as a critical mechanism of central sensitization in AR. In fact, microglial NLRP3 inflammasome activation has been verified to be associated with central sensitization in chronic headache disorders [24,26]. Recently, Liu et al. [37] found that blocking the NLRP3/IL-1β axis in microglia alleviated spinal neuron activation and OVA-induced chronic pruritus. These findings highlight that NLRP3 inflammasome activation is a critical molecular step underlying neuronal hyperexcitability in various pathological conditions.

There is increasing evidence indicating that peripheral immune mediators in chronic inflammatory diseases have the capacity to activate microglia and induce neuroinflammation [27,28]. Many scholars have hypothesized that peripheral immune signals in AR can cross the BBB to induce neuroinflammation in the brain [10,38]. This study first identified evidence of BBB damage in the TNC of AR mice. This means that peripheral cytokines in AR mice may enter the TNC to mediate neuroinflammation through increased BBB permeability. AR is characterized by type 2 immune overactivation with significantly elevated levels of type 2 cytokines like IL-4, IL-5, and IL-13 in the peripheral blood. We found that the IL-4 concentrations in the TNC tissues of AR mice were markedly higher than those in controls, without significant changes in the mRNA levels of the IL-4 gene. Moreover, our findings reveal a correlation between IL-4 levels in the peripheral circulation and those in the TNC of AR mice. These experimental data imply that IL-4 may be involved in microglial NLRP3 inflammasome activation in AR mice. However, our data showed that IL-4 stimulation alone does not induce microglial NLRP3 inflammasome activation in vitro. Traditionally, IL-4 has long been considered an anti-inflammatory cytokine in the brain, and its canonical role is to inhibit microglia-mediated neuroinflammation [39]. Therefore, we further analyzed the transcriptomic data of primary microglia after IL-4 stimulation. The findings indicated that IL-4-stimulated microglia exhibited the features of “microglia priming”, a recognized microglia phenotype with increased pro-inflammatory potential. Furthermore, IL-4 exposure promoted LPS-induced NLRP3 inflammasome activation in microglia. These results preliminarily indicate that IL-4 may significantly enhance the pro-inflammatory tendency of microglia, thereby mediating NLRP3 inflammasome activation. Several studies have reported this similar role of IL-4 in macrophages. Czimmerer et al. [40] found that IL-4 priming created a hyperinflammatory gene expression program in macrophages in response to LPS stimulation through epigenetic reprogramming. Moreover, Dang et al. [41] demonstrated that IL-4 pretreatment aggravated the inflammatory response of macrophages to LPS by activating the glycolysis pathway. Microglia are considered to be resident macrophages in the CNS [42] and share similarities with macrophages in terms of gene expression and phenotypic plasticity [43]. Therefore, this noncanonical pro-inflammatory role of IL-4 in microglia seems reasonable. Indeed, evidence has emerged that IL-4 promotes microglial neuroinflammation under certain pathological conditions. Wang et al. [44] found that neonatal exposure to IL-4 resulted in increased numbers of microglia and neuroinflammatory damage in the rat hippocampus. Our study revealed that blockade of the IL-4 signaling pathway inhibits NLRP3 inflammasome activation in microglia. These results provide further evidence that IL-4, a key mediator of allergic inflammation, is important in driving microglial neuroinflammation in AR mice. Collectively, IL-4 exhibits a multifaceted regulatory capacity in neuroimmune communication and may promote neuroinflammation within Th2-skewed microenvironments. Moreover, inhibition of IL-4 improved the central sensitization of the TNC and reversed pathological changes in AR mice. This suggests not only that IL-4 is a central mediator in allergic inflammation, but that it is also a key information carrier in the immune–brain communication circuitry in AR. This study also revealed elevated IL-13 levels in the TNC of AR mice. As is well established, both IL-4 and IL-13 exert their biological effects by signaling through the shared receptor chain IL-4 Rα. Given the expression of IL-4 Rα on microglia, IL-13 may exert effects analogous to those of IL-4 on these cells. Existing research indicates that IL-13 priming similarly enhances LPS-induced inflammation in macrophages [41]. However, the role of IL-13 in mediating neuroinflammation and central sensitization in AR warrants further investigation.

Our findings hold clinical translational potential, as targeting neuroimmune dysregulation in the CNS represents a promising therapeutic strategy for AR. Notably, intranasal administration offers a highly promising noninvasive delivery route, directly transporting therapeutics to the olfactory bulb, brain stem, and other CNS regions via olfactory and trigeminal pathways [45]. Moreover, the selection of drug carriers is paramount. Nanocarriers demonstrate multifaceted advantages, including low toxicity, high drug-loading capacity, and enhanced delivery efficiency, that have established their pivotal role in brain-targeted drug delivery [46]. Current consensus recognizes the combination of intranasal delivery with nanocarriers as one of the most promising strategies for transporting macromolecules to the CNS [45]. Therefore, the targeted CNS delivery of therapeutics such as IL-4 Rα antagonists through intranasal nanocarriers may emerge as a valuable treatment option for AR in the future.

This study has several limitations. First, the precise mechanism by which central sensitization leads to imbalance of autonomic and immune functions is unknown. Second, cooperative factors likely interact with IL-4 to drive NLRP3 inflammasome activation in microglia in AR, and these synergistic mechanisms are not fully understood. Third, although both female and male mice are widely used for AR model construction, it is necessary to consider the effect of sex factors on microglia. Fourth, cannula implantation is an invasive approach that may induce microglial activation. Therefore, future studies should explore noninvasive tools for the targeted modulation of microglia.

## Conclusion

In summary, we revealed a previously unknown mechanism by which microglia promote central sensitization in the TNC by activating the NLRP3/IL-1β axis, thereby contributing to the pathogenesis of AR. Strategies to reverse this dysfunctional central plasticity might represent a promising new avenue for AR therapy. Furthermore, the present findings underscore the neglected noncanonical pro-inflammatory role of IL-4 in microglia and broaden our understanding of the neuroimmune mechanisms in allergic diseases.

## Materials and Methods

### Animals

Male C57BL/6 mice aged 6 to 8 weeks were acquired from Shulaibao Biotech (Wuhan, China). The animals were maintained under the specific-pathogen-free conditions at the Experimental Animal Center of Renmin Hospital of Wuhan University, with environmental parameters strictly controlled (humidity, 50% ± 5%; temperature, 23 ± 1 °C; and 12-h light/dark cycle). They were permitted 1 week to acclimate before the experiments commenced. Following a 7-d acclimatization period prior to experimentation, all procedures complied with the ARRIVE guidelines for laboratory animal welfare. This study protocol received formal ethical approval (Ethics Committee of Renmin Hospital of Wuhan University, Approval No. WDRM20190419).

### AR model establishment

The AR murine model was developed following established protocols [23,47]. Initial sensitization involved administering 3 intraperitoneal injections (days 0/7/14) containing 100 μg of OVA (Sigma, USA) combined with 5 mg of aluminum hydroxide in 300 μl of phosphate-buffered saline (PBS) solution, while control groups received equivalent PBS volumes. Subsequent nasal provocation commenced on day 21, with animals receiving bilateral intranasal instillations (10 μl per nostril) of OVA solution (200 μg in 20 μl of PBS) daily for 14 consecutive days. To assess AR manifestations, 2 blinded investigators counted sneezes and nasal rubbing episodes in mice over a 10-min period starting 15 min after the final OVA challenge on day 34.

### Whole-cell patch-clamp recordings

Following deep anesthesia induction, experimental animals received transcardial perfusion with ice-cold oxygenated *N*-methyl-d-glucamine (NMDG)-based slicing solution. The solution pH was meticulously buffered to 7.35 using additions of NMDG or HCl. Following rapid dissection, brain stem tissue was promptly submerged into ice-cold oxygenated NMDG-based slicing solution. Using a vibratome (Leica, Germany), 200-μm coronal brain stem sections were prepared. Then, they were subjected to 2-stage incubation: primary recovery in NMDG cutting solution (34 °C, 10 min) followed by maintaining in oxygenated artificial cerebrospinal fluid (25 °C, 1 h). For electrophysiological analysis, prepared slices were positioned in a perfusion chamber with continuous oxygenated artificial cerebrospinal fluid flow (2 to 3 ml/min) and visualized with a microscope featuring infrared differential interference contrast (Olympus, Japan). Neurons from lamina I/II in the TNC were identified and recorded using whole-cell patch-clamp recordings. Current-evoked APs were induced using 2-s depolarizing pulses ranging from 20 to 120 pA in increments of 20 pA, with trials spaced 30 s apart. The AP frequency was calculated as the total number of spikes per stimulus duration. Resting membrane potential measurements were initiated within 60 s after achieving whole-cell configuration. The membrane potential at which the phase plot slope exceeded 15 mV/ms was denoted as the threshold. The first spike latency was the time duration between the onset of the stimulus and the first spike. The first interspike interval was calculated as the time interval between the first and second APs. Electrophysiological data were captured using a MultiClamp 700B amplifier (Molecular Devices, USA), filtered at 2 kHz, and acquired at 10 kHz via the Digidata 1440A interface (Molecular Devices, USA) with data acquisition handled by Clampex 10.2. Only data with series resistance variations ≤15% from baseline values (15 to 25 Ω) were included in the analysis.

### Stereotaxic microinjection and chemogenetic experiments

Mice were anesthetized with pentobarbital sodium (60 mg/kg) and secured in stereotactic apparatus (RWD Life Sciences, China) for targeted viral delivery. Bilateral microinjections (0.2 μl/side) of chemogenetic vectors rAAV-hSyn-hM4D(Gi)-EGFP (5.15 × 10^12^ viral genomes/ml) (BrainVTA Technology, China) were delivered into the TNC of AR mice at 0.1 μl/min infusion rates using Hamilton syringes with 33-G needles. Control animals received equivalent doses of EGFP-expressing control vectors. Stereotaxic coordinates relative to bregma, as referenced from a mouse brain atlas [48], were set as follows: anterior–posterior, 7.70 mm; medial–lateral, ±1.76 mm; dorsal–ventral, −4.75 mm. Chemogenetic inhibition was initiated by intraperitoneal administration of 5 mg/kg clozapine-*N*-oxide (MedChemExpress, USA) 30 min prior to OVA challenge.

### Intracerebral drug delivery

To delineate the pathophysiological involvement of microglial activation in AR-related central sensitization, pharmacological suppression of microglia was achieved using minocycline (MedChemExpress, USA). In anesthetized mice immobilized in a stereotaxic apparatus, a catheter (0.25-mm diameter, RWD Life Sciences) was chronically implanted in the TNC. All mice that underwent cannula implantation surgery were subjected to subsequent experiments following a 7-d recovery period. The cannula was solidly cemented into the skull of the mouse and capped until the drug was administered. A microinjection system consisting of an infusion pump, a 10-μl Hamilton syringe, and an internal injector was coupled to the guide cannula. Bilateral TNC regions received minocycline infusions in PBS (10 mg/ml, 100 nl/side) administered at 100 nl/min. The injector was slowly taken out 5 min following infusion. Minocycline treatment was administered 1 h prior to daily OVA challenge for 14 d. The dose of minocycline used in this study is based on previous reports, wherein it effectively mitigated microglial activation [49].

To block the IL-4 signaling pathway, bilateral injections targeting the TNC were performed using either IL-4 NAb (1 μg/μl, 0.2 μl/side) (R&D Systems, USA) or mouse IgG isotype control. The steps for intracerebral administration were described above. The dose of IL-4 NAb used is based on previous reports, wherein it effectively inhibited microglial inflammation [50]. Mice were injected with IL-4 NAb 1 h prior to OVA challenge, with injections administered on day 21 and day 27.

### EB assay

The EB assay was employed for evaluating BBB integrity, as previously reported [51]. Briefly, mice were given an injection of 2% EB (Sigma, USA) solution (4 ml/kg) via the tail vein. Following a 24-h circulation period, mice underwent anesthesia and transcardial perfusion using 20 ml of PBS. Subsequently, brain tissues were collected, and brain images were acquired using a digital camera (Olympus, Japan). Confocal microscopy (Olympus, Japan) was employed to observe the TNC slices. The concentration of EB in TNC tissues was quantified by spectrophotometry at 610 nm and calculated from an EB standard curve.

### Cell culture and treatments

The murine microglial cell line BV-2 and the murine neuronal cell line Neuro-2a were obtained from Procell Biotech (Wuhan, China). Primary neonatal microglia were isolated from neonatal C57BL/6 mice at postnatal day 0 using an enzymatic dissociation approach as previously described [52]. Cells were maintained in Dulbecco’s modified Eagle’s medium (Gibco, USA) supplemented with 10% fetal bovine serum (Gibco, USA) and 1% penicillin/streptomycin (Solarbio, China) andincubated at 37 °C in an atmosphere enriched with 5% CO_2_. The possible pro-inflammatory effects of IL-4 on microglia were assessed by pretreating both primary neonatal microglia and BV-2 cells with IL-4 (20 ng/ml) for 24 h, followed by a 24-h stimulation with LPS at 1 μg/ml. Following this, cells were collected to conduct subsequent experiments.

### Microglia–neuron co-culture

To explore how microglial NLRP3 inflammasome activation affects AR-related central sensitization in vitro, we isolated primary adult microglia from the brain stem of AR or control mice using magnetic-activated cell sorting technology, as previously reported [42]. To inhibit the activation of the NLRP3 inflammasome, primary adult microglia were exposed to 10 μM MCC950 (MedChemExpress, USA), a specific NLRP3 inflammasome inhibitor, for 24 h. Briefly, the upper chamber of a Transwell 24-well plate (Corning, USA) was seeded with treated primary adult microglia (2 × 10^4^ cells/well), while Neuro-2a cells (2 × 10^5^ cells/well) were seeded into the lower chamber. Following a 24-h co-culture period, Neuro-2a cells were harvested for further experiments.

### Enzyme-linked immunosorbent assay

Following anesthesia, mice underwent tracheotomy. A catheter was inserted through the upper airway into the nasopharynx, and 1 ml of ice-cold PBS was gently infused. NLF was collected at the nostrils. The concentrations of Th1/Th2 cytokines (IFN-γ, IL-4, IL-5, and IL-13) in NLF were quantified via enzyme-linked immunosorbent assay (ELISA) kits (R&D Systems) following the manufacturer’s instructions. In addition, IL-4 levels in peripheral blood and the TNC were also measured using the ELISA kit (R&D Systems). Using a microplate reader, absorbance was detected at 450 nm.

### Histological analysis

Following anesthesia, mice were euthanized, and their noses were obtained. The nasal tissues were fixed in 4% paraformaldehyde (PFA) for 24 h, soaked in 10% ethylenediamine tetraacetic acid decalcification solution for 2 weeks, embedded in paraffin, sectioned into 4-μm-thick slices, and stained with hematoxylin–eosin (to visualize eosinophils) and periodic acid–Schiff stain (to identify goblet cells). Eosinophils and goblet cells were quantified in 3 randomly chosen high-power fields. Quantification was performed by 2 independent researchers blinded to experiments.

### Immunofluorescence staining

Following anesthesia, experimental subjects underwent transcardial perfusion with cold PBS. Brain tissues were rapidly excised and subjected to sequential processing: primary fixation (4% PFA, 4 °C, 24 h), dehydration through graded sucrose immersions (20% → 30%, 24 h each at 4 °C), freezing, and final embedding optimum cutting temperature medium. TNC tissues were collected, sliced into 10-μm-thick sections based on the mouse brain atlas, and subjected to overnight incubation at 4 °C with primary antibodies, including anti-c-Fos (1:100, Abcam), anti-p-ERK (1:100, ABclonal), anti-Iba-1 (1:100, Abcam), anti-CD68 (1:100, CST), anti-NLRP3 (1:100, Abcam), and anti-IL-1β (1:100, CST), followed by 2-h incubation with fluorophore-conjugated secondary antibodies at room temperature.

Nose sections were exposed to primary antibodies targeting the following proteins: TH (1:100, Servicebio), NPY (1:50, Santa Cruz), ChAT (1:100, Servicebio), and VIP (1:100, Servicebio), during an overnight incubation at 4 °C. Subsequently, they were probed with appropriate fluorophore-conjugated secondary antibodies.

For immunocytochemistry, the cultured cells were washed with cold PBS, fixed in 4% PFA for 30 min, permeabilized with 0.2% Triton X-100, and blocked using 10% goat serum at room temperature for 1 h. Primary antibodies, including anti-Iba-1 (1:100, Abcam), anti-CD68 (1:100, CST), anti-NLRP3 (1:100, Abcam), anti-NF-κB p65 (1:500, CST), anti-IL-1β (1:100, CST), anti-c-Fos (1:200, Abcam), anti-p-ERK (1:100, ABclonal), anti-phosphorylated NMDAR 2B subunit (anti-p-NR2B; 1:200, Abcam), and anti-phosphorylated AMPAR GluA1 subunit (anti-p-GluA1; 1:200, Abcam), were applied and incubated overnight at 4 °C, followed by incubation with secondary antibodies.

4′,6-Diamidino-2-phenylindole was used to stain nuclei at room temperature for 10 min. Images were obtained using a fluorescence microscope and analyzed by the ImageJ software (National Institutes of Health, USA). Mean fluorescence intensity was calculated from 3 randomly selected microscopic fields from each section for each animal. A total of 3 animals per group were analyzed. Image quantification was performed by 2 independent researchers blinded to experiments.

### Quantitative real-time PCR

TRIzol reagent (Servicebio, China) was employed to obtain total RNA from TNC tissues. RNA purity and concentration were determined by a spectrophotometer. Then, PrimeScript RT Reagent Kit (Takara, Japan) was employed to reverse-transcribe total RNA into complementary DNA. qRT-PCR was executed on a real-time PCR device (Bio-Rad, USA) with the SYBR Green RT-PCR kit (Takara, Japan) following the manufacturer’s protocol. The primer sequences for GAPDH are as follows: forward, 5′-CCAGCTCGTCCTGTAGACAA-3′; reverse, 5′-GCCTTGACTGTGCCGTTGA-3′; and those for IL-4 amplification are as follows: forward, 5′-CTCGAATGTACCAGGAGCCA-3′; reverse, 5′-TGTGGTGTTCTTCGTTGCTG-3′. The levels of mRNA relative to GAPDH were normalized with the 2^−ΔΔCT^ method.

### Western blotting

Cells or TNC tissues were homogenized and lysed in radioimmunoprecipitation assay buffer (Servicebio, China) incorporated with a phosphatase inhibitor cocktail and phenylmethylsulfonyl fluoride (Servicebio, China). Sodium dodecyl sulfate–polyacrylamide gel electrophoresis was used to separate equal protein loads, which were then transferred to polyvinylidene fluoride membranes (Millipore, USA). Following a 2-h block with 5% bovine serum albumin (Sigma, USA) at room temperature, the membranes were incubated with primary antibodies, including anti-c-Fos (1:1,000, Abcam), anti-p-ERK (1:1,000, ABclonal), anti-NR2B (1:1,000, Abcam), anti-p-NR2B (1:1,000, Abcam), anti-GluA1 (1:1,000, Abcam), anti-p-GluA1 (1:1,000, Abcam), anti-NLRP3 (1:1,000, Abcam), anti-NF-κB p65 (1:1,000, CST), anti-IL-1β (1:1,000, CST), anti-CD68 (1:1,000, CST), and anti-GAPDH (1:1,000, Proteintech), at 4 °C overnight. After washing with PBS, the membranes were exposed to the corresponding horseradish peroxidase-conjugated secondary antibody (1:5,000, Proteintech). All membranes were visualized through an imaging system (Bio-Rad, USA) with enhanced chemiluminescence reagent kits (Servicebio, China). Densitometric analysis of protein bands was performed using ImageJ, normalized to the expression of GAPDH.

### Bioinformatics analysis

The transcriptomic profiles of primary microglia after IL-4 stimulation were analyzed using a bioinformatics approach.

#### Differential gene expression analysis

Using the “limma” package (v.3.58.1), DEGs were detected in the GSE157891 datasets [53]. The selection criteria for DEGs were *P* < 0.05 and |log_2_ FC| > 0.585. The “ggplot2” package (v.3.5.1) was then employed to plot volcano diagrams that depicted the expression patterns of DEGs.

#### Functional enrichment analysis

In order to comprehend the functional annotation of DEGs, the “clusterProfiler” package (v4.10.0) was used to execute GO and KEGG pathway enrichment analysis, with statistical significance criteria established at *P* < 0.05. Furthermore, gene set enrichment analysis was executed on pathways of interest by the “clusterProfiler” package (v4.10.0).

### Statistical analyses

For statistical analyses and graph plotting, GraphPad Prism (v8.0, GraphPad Software Inc., USA) was employed. All data are expressed as mean ± standard deviation. Comparisons between 2 groups were made using Student *t* tests, whereas one-way analysis of variance and Tukey’s post hoc tests served for multigroup comparisons. A *P* value <0.05 was regarded as statistically significant.

## Supplementary Material

20250925-1

## Data Availability

All data generated or analyzed during this study are included in this paper. Further inquiries can be directed to the corresponding author.
