# Erratum to “Interleukin-4-Mediated NLRP3 Inflammasome Activation in Microglia Contributes to Allergic Rhinitis via Central Sensitization”

**DOI:** 10.34133/research.1066

**Published:** 2026-01-14

**Authors:** Hao Lv, Yunfei Wang, Lu Tan, Yulie Xie, Peiqiang Liu, Mengting Guan, Jianchao Cong, Yu Xu

**Affiliations:** ^1^ Department of Otolaryngology-Head and Neck Surgery, Renmin Hospital of Wuhan University, Wuhan, China.; ^2^ Department of Rhinology and Allergy, Renmin Hospital of Wuhan University, Wuhan, China;; ^3^ Research Institute of Otolaryngology-Head and Neck Surgery, Renmin Hospital of Wuhan University, China.; ^4^ Hubei Province Key Laboratory of Allergy and Immunology, Wuhan, China.

In the Research Article “Interleukin-4-Mediated NLRP3 Inflammasome Activation in Microglia Contributes to Allergic Rhinitis via Central Sensitization ” [1], the authors identified several inadvertent errors during a post-publication review. Specifically, the image labeled “DAPI” in Control group in Fig. 3E was inadvertently misused as the image labeled “DAPI” in Control group in Fig. 3D. The immunofluorescence images in IL-4 group in Fig. 8D were mistakenly placed in the LPS group panel of Fig. 8B. These errors were due to unintentional oversights during figure assembly. Moreover, the immunofluorescence images in Control group in Fig. 8D, the H&E image in IL-4 NAb group in Fig. 10C, and the immunofluorescence image in EGFP group in Fig. S7 were inadvertently submitted in incorrect versions. These unintentional errors originated from inadequate file management and a lapse in manual verification during image selection. All identified issues are technical oversights in nature and do not affect the results or conclusions of the paper. The authors regret these inadvertent errors and sincerely apologize for any inconvenience caused.

**Fig. 3.**
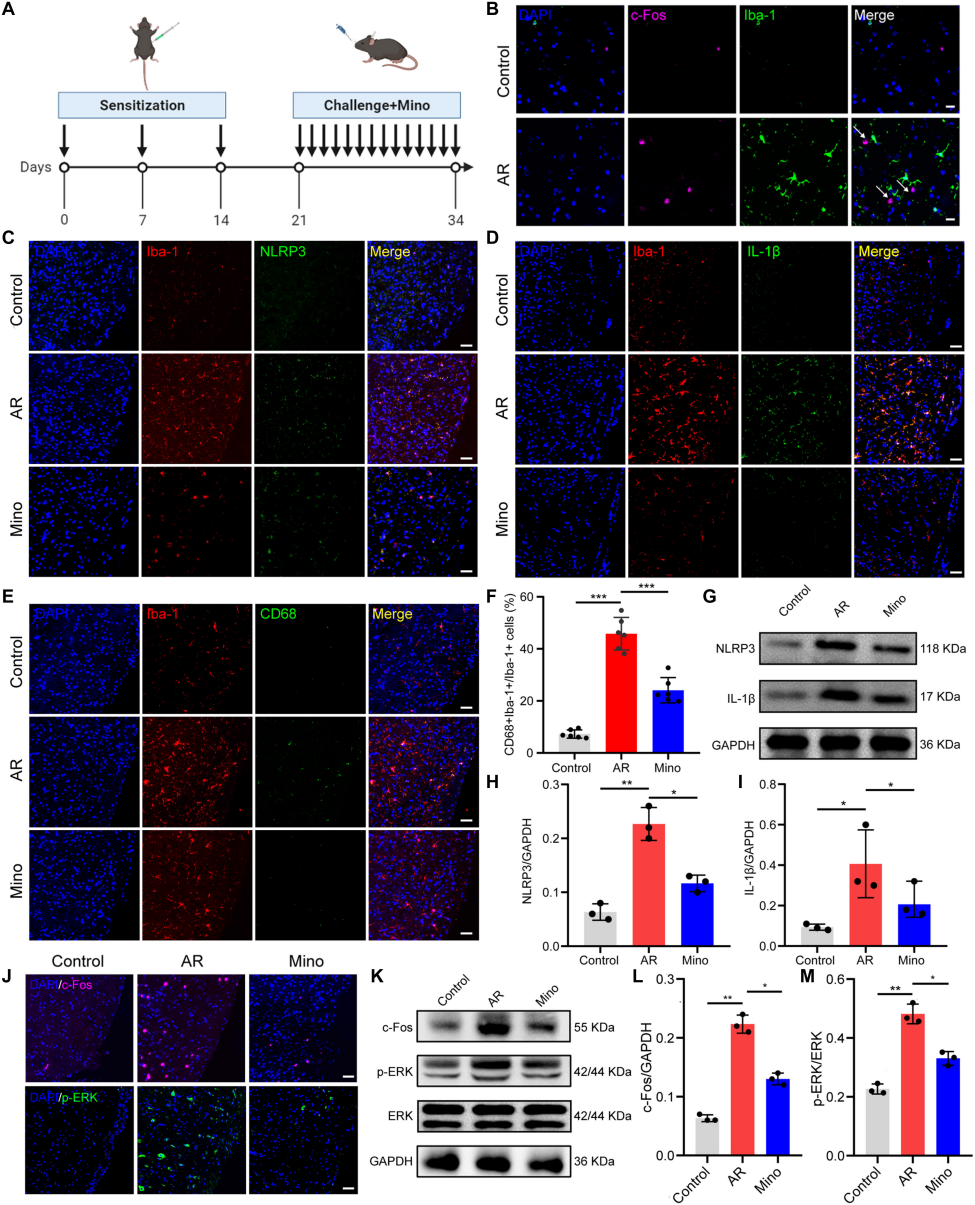
Inhibition of microglia activation attenuated NOD-like receptor protein 3 (NLRP3) signaling and central sensitization in AR. (A) Schematic diagram of the experimental design. (B) Double immunofluorescence staining for c-Fos and Iba-1. Scale bars, 20 μm. (C) Double immunofluorescence staining for Iba-1 and NLRP3. Scale bars, 50 μm. (D) Double immunofluorescence staining for Iba-1 and IL-1β. Scale bars, 50 μm. (E) Double immunofluorescence staining for Iba-1 and CD68. Scale bars, 50 μm. (F) Quantification of the percentage of CD68-positive microglia. N = 6 mice per group. (G) Western blotting for the detection of NLRP3 and IL-1β. (H and I) Quantification analysis of the protein expression of NLRP3 and IL-1β. N = 3 mice per group. (J) Immunofluorescence staining with c-Fos and p-ERK in the TNC. Scale bars, 50 μm. (K) Western blotting for the detection of c-Fos and p-ERK of TNC tissues. (L and M) Quantification analysis of c-Fos and p-ERK protein expression. N = 3 mice per group. *P < 0.05; **P < 0.01; ***P < 0.001. Mino, minocycline.

**Fig. 8.**
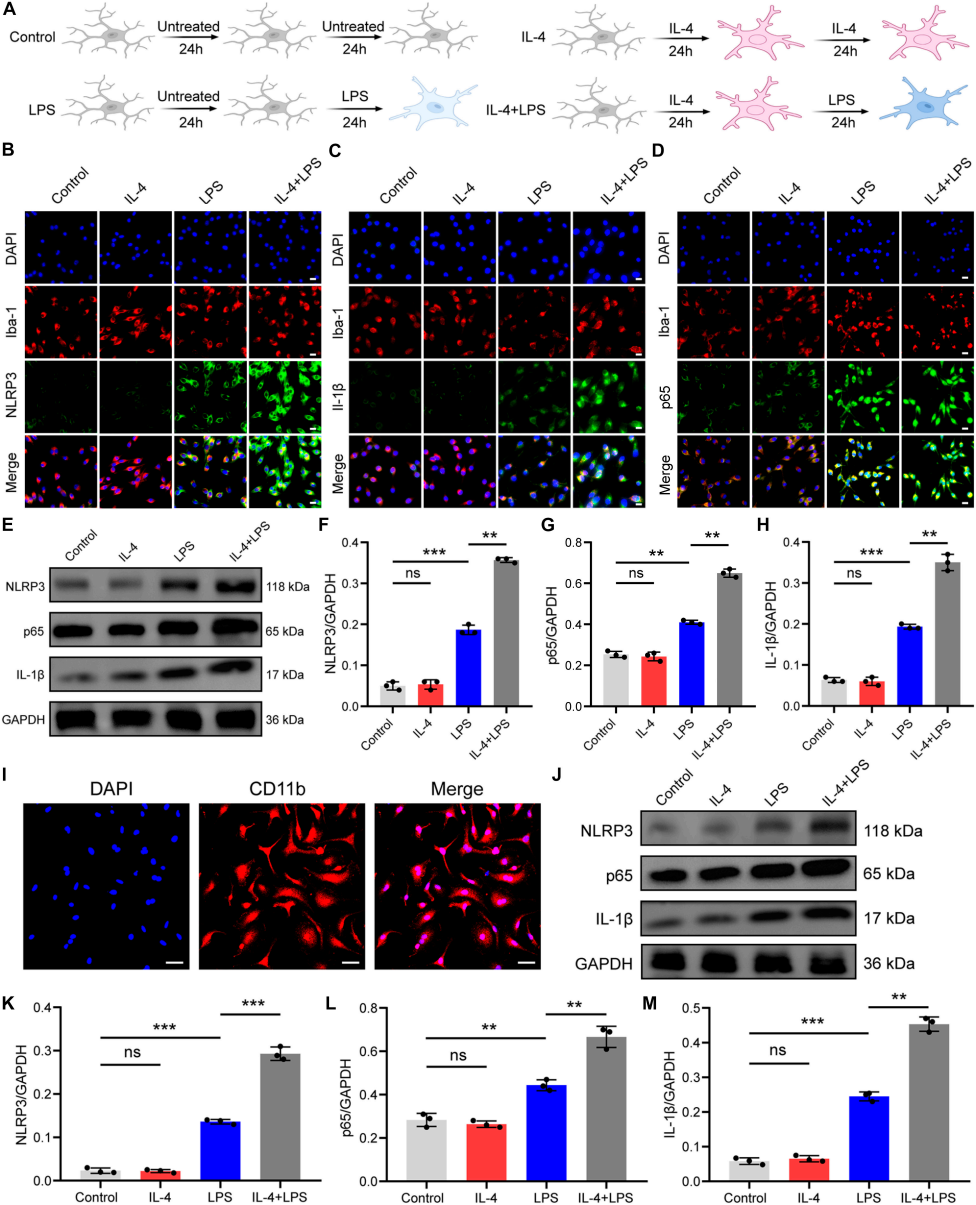
IL-4 exposure enhanced lipopolysaccharide (LPS)-induced NLRP3 signaling in microglia. (A) Flowchart of experimental design. (B) Double immunofluorescence staining for Iba-1 and NLRP3 in BV-2 cells. Scale bars, 20 μm. (C) Double immunofluorescence staining for Iba-1 and IL-1β in BV-2 cells. Scale bars, 20 μm. (D) Double immunofluorescence staining for Iba-1 and p65 in BV-2 cells. Scale bars, 20 μm. (E) Western blotting analysis of NLRP3, IL-1β, and p65 expression in BV-2 cells. (F to H) Quantification analysis of NLRP3, IL-1β, and p65 expression. (I) Immunofluorescent staining of CD11b in primary mouse microglia. (J) Western blotting analysis of NLRP3, IL-1β, and p65 expression in primary mouse microglia. (K to M) Quantification analysis of NLRP3, IL-1β, and p65 expression. *P < 0.05; **P < 0.01; ***P < 0.001.

**Fig. 10.**
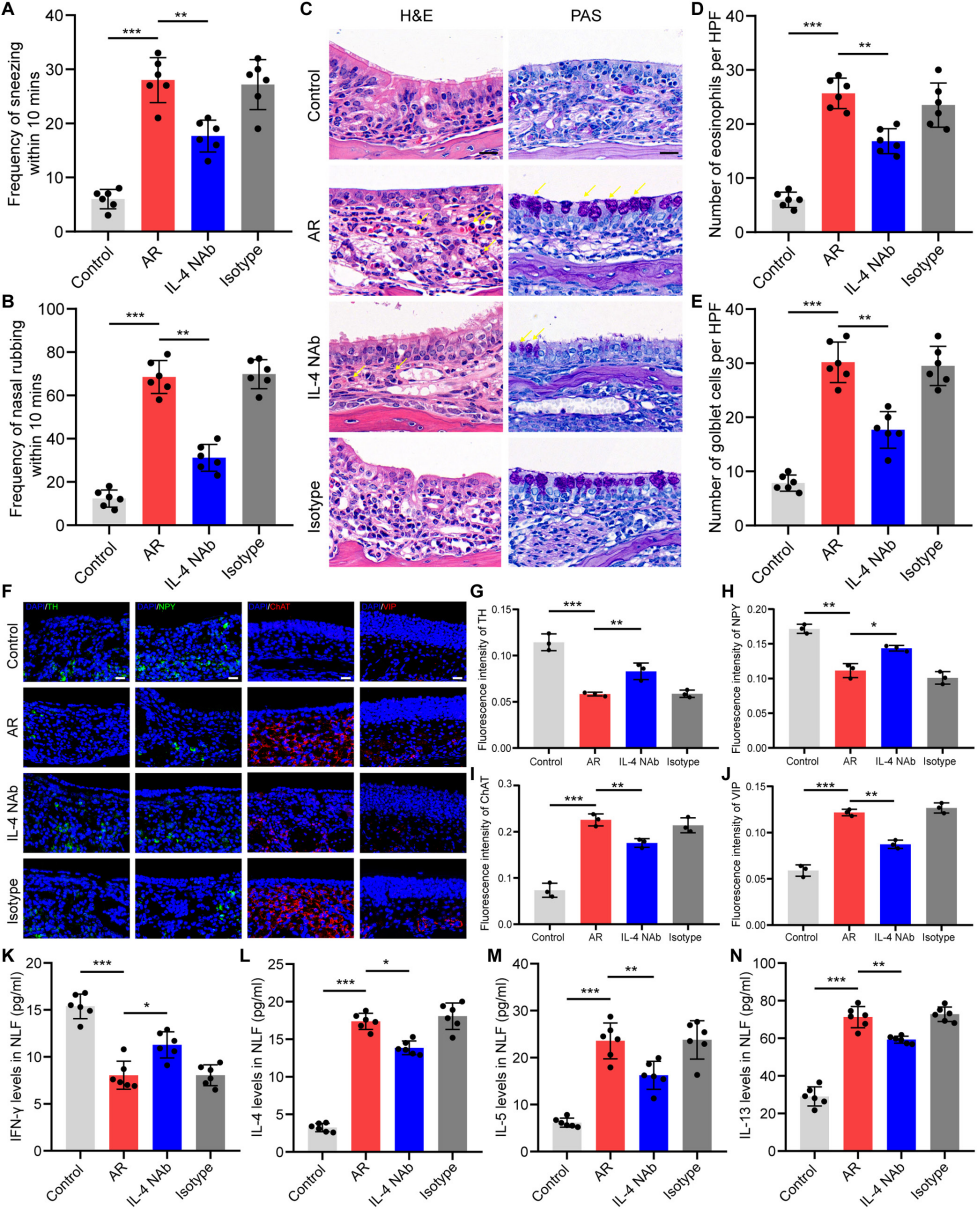
Intracerebral injection of IL-4 NAbs improved AR. (A and B) Quantification of nasal rubbing and sneezing episodes within 10 min. N = 6 mice per group. (C) H&E and PAS staining of nose sections. Scale bars, 20 μm. (D and E) Quantification of eosinophil and goblet cell counts in a ×400 high-power field. N = 6 mice per group. (F) Immunofluorescence staining with sympathetic nerve markers (TH and NPY) and parasympathetic nerve markers (ChAT and VIP). Scale bars, 20 μm. (G to J) Quantification of the immunofluorescence intensity of TH, NPY, ChAT, VIP. N = 3 mice per group. (K to N) Content of IFN-γ, IL-4, IL-5, and IL-13 in the NLF. N = 6 mice per group. *P < 0.05; **P < 0.01; ***P < 0.001.
