# Supplementary material for: Interleukin-4-Mediated NLRP3 Inflammasome Activation in Microglia Contributes to Allergic Rhinitis via Central Sensitization
Source: Research (Wash D C). 2025 Sep 25;8:0897. doi: 10.34133/research.0897 (PMC12463543; doi:10.34133/research.0897)
Supplement: Supplementary 1 — Figs. S1 to S7 [file research.0897.f1.docx]

**
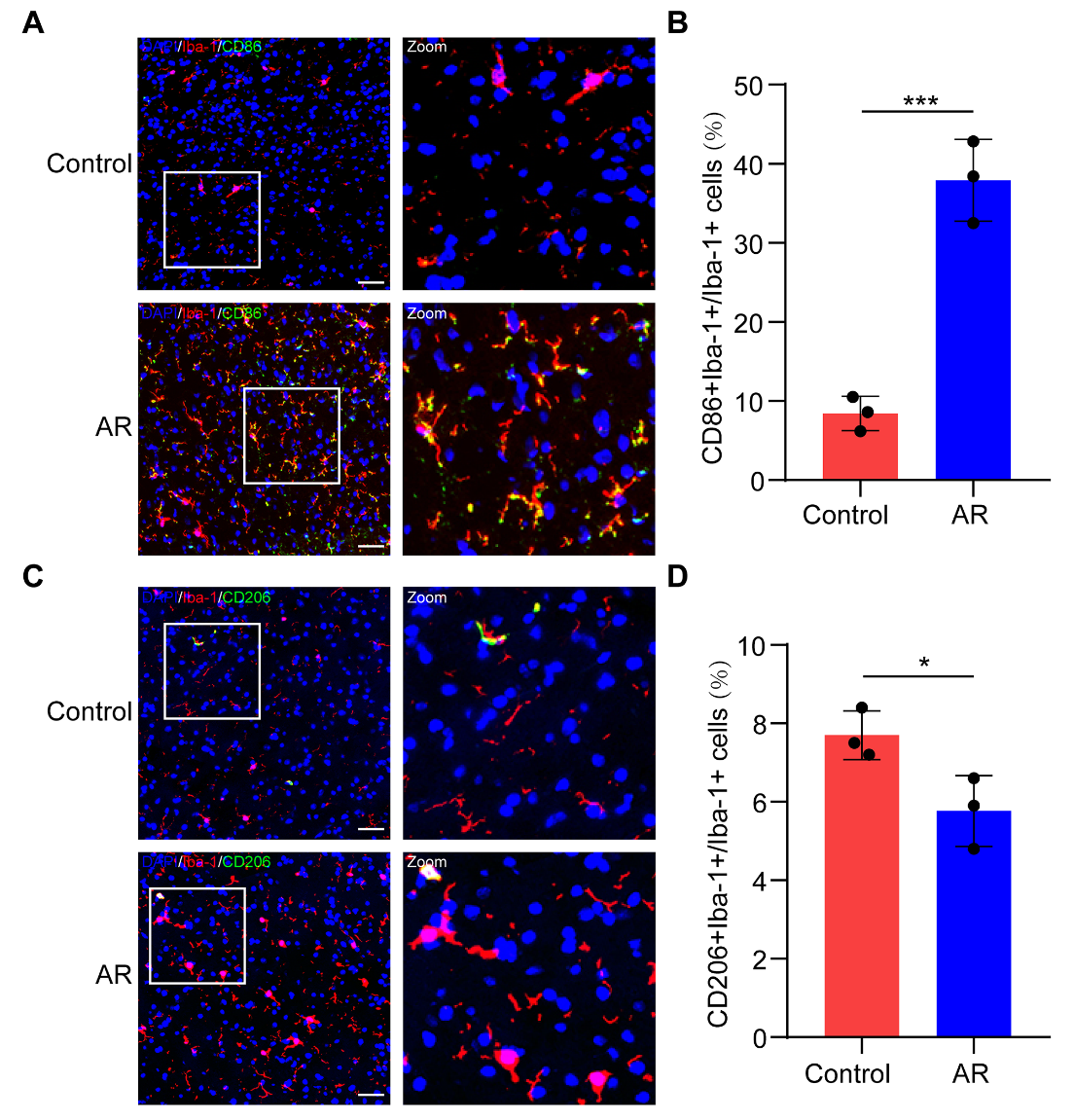
**

Figure S1. Characterization of M1/M2 microglial polarization in the TNC. (A) Double immunofluorescence staining for Iba-1 and CD86. (B) Quantitative analysis of the percentage of CD86-positive microglia. (C) Double immunofluorescence staining for Iba-1 and CD206. (D) Quantitative analysis of the percentage of CD206-positive microglia. Scale bars, 50 μm. N = 3 mice per group. *p < 0.05, ***p < 0.001.


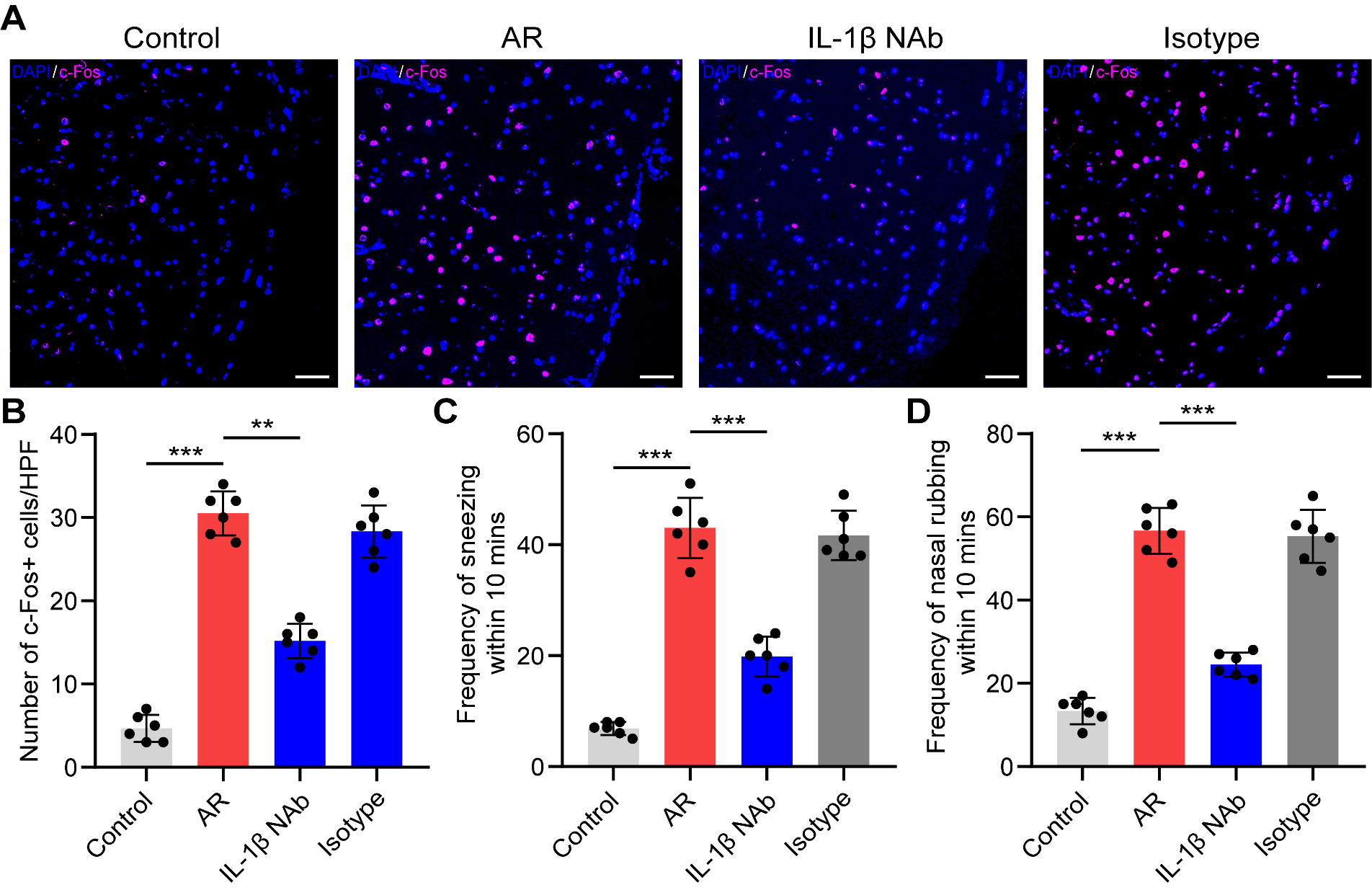


Figure S2. Intracerebral injection of IL-1β neutralizing antibodies improved central sensitization and AR symptoms. (A) Immunofluorescence staining for c-Fos. (B) Quantification of c-Fos^+^ cells in the TNC. N = 3 mice per group. (C) Quantification of nasal rubbing and sneezing episodes within 10 minutes. Scale bars, 50 μm. N = 6 mice per group. **p < 0.01, ***p < 0.001.


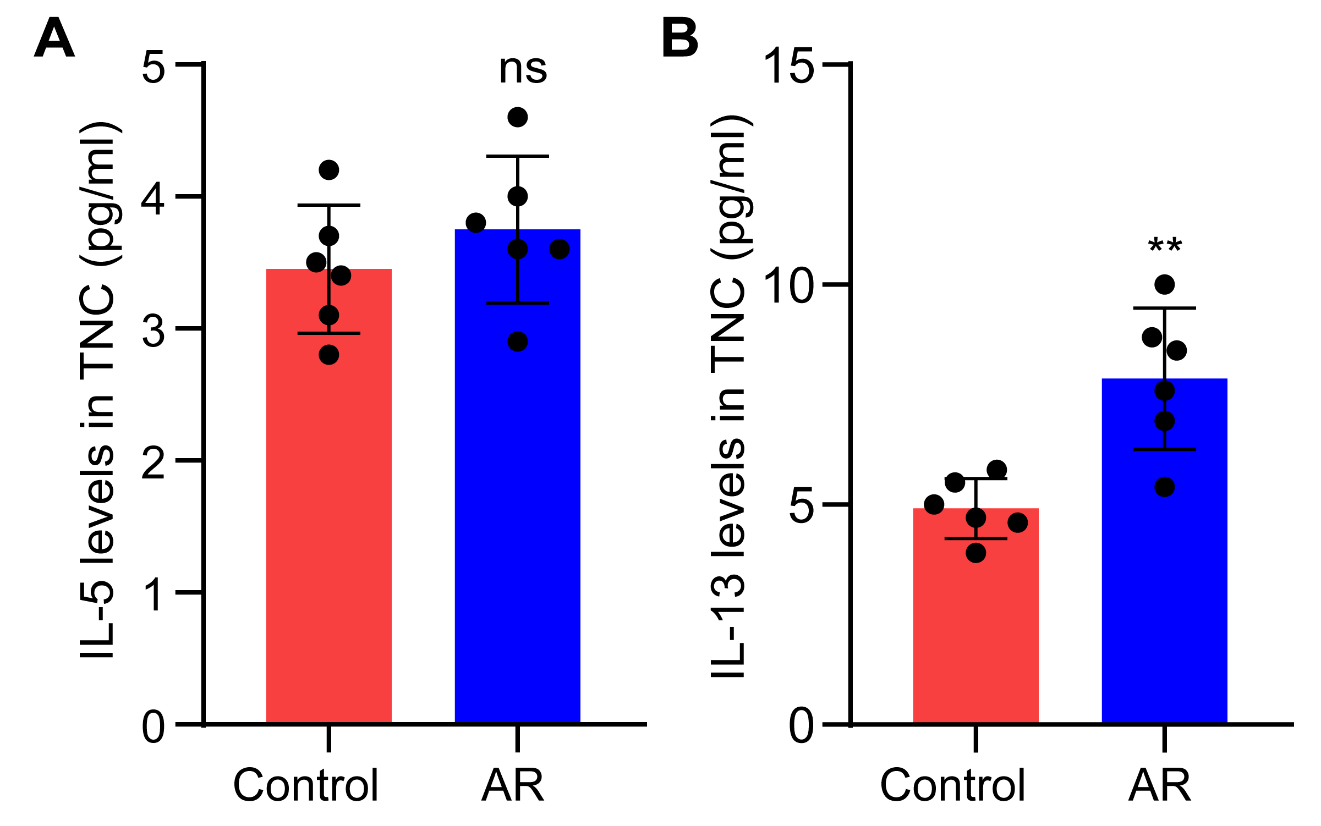


Figure S3. Type 2 cytokine expression in the TNC of AR mice. (A) IL-4 levels in the TNC. (B) IL-13 levels in the TNC. N = 6 mice per group. **p < 0.01.


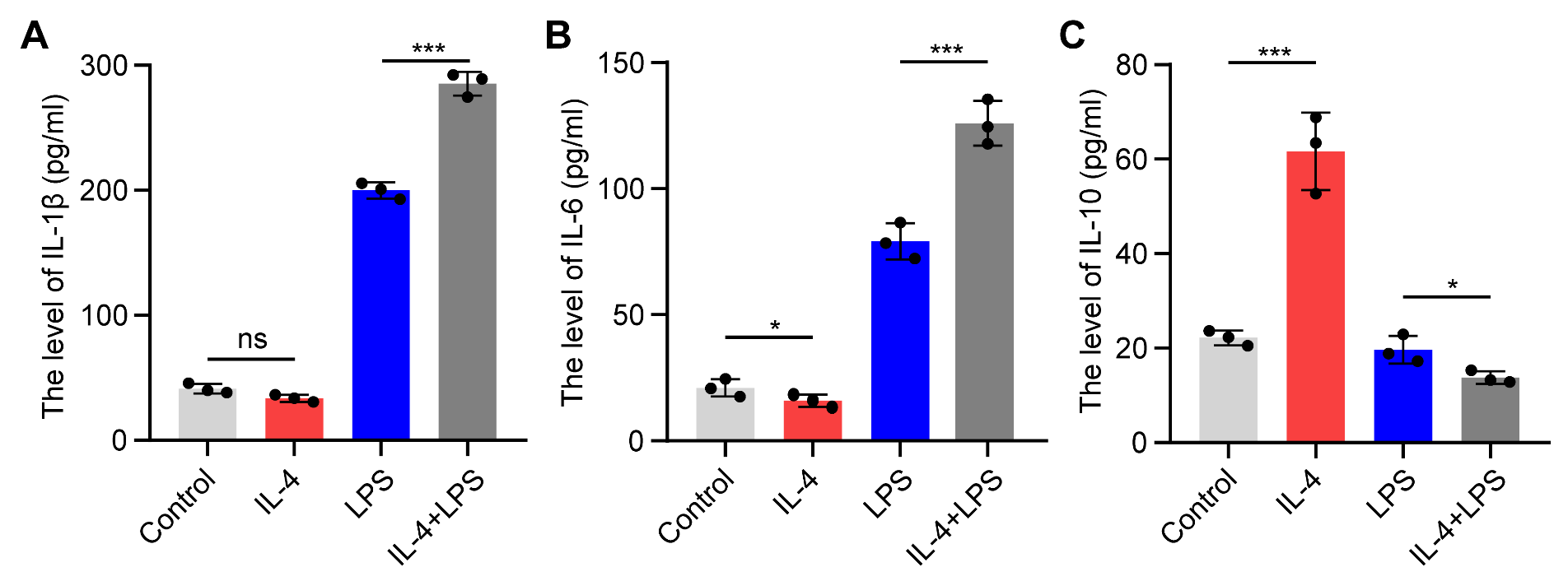


Figure S4. IL-4 priming increased LPS-induced M1-associated cytokines of BV2 cells. (A) IL-1β levels in the supernatant. (B) IL-6 Levels in the supernatant. (C) IL-10 levels in the supernatant. *p < 0.05, ***p < 0.001.


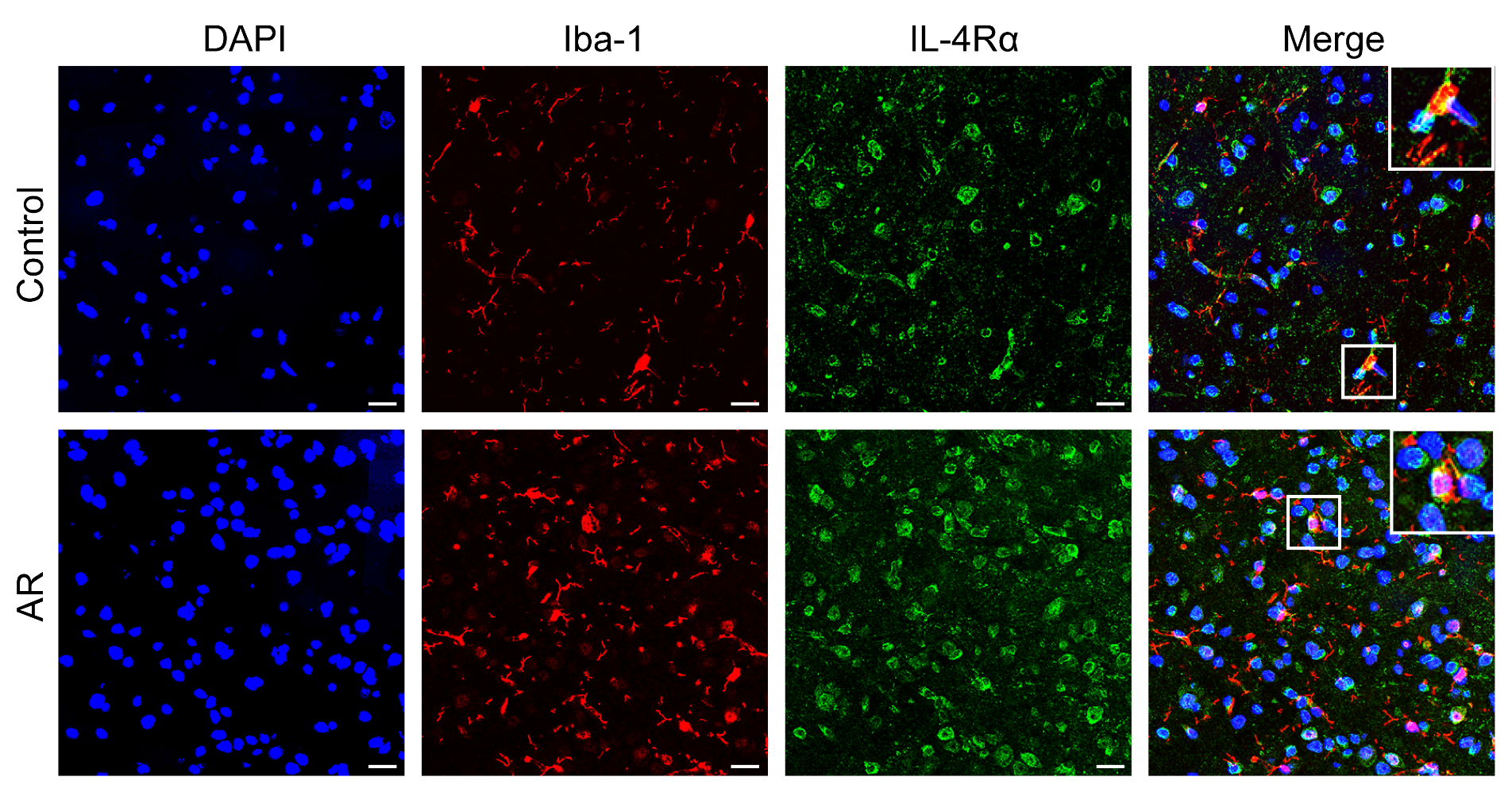


Figure S5. IL-4 Rα expression on microglia in the TNC of mice. Double immunofluorescence staining for Iba-1 and IL-4 Rα. Scale bars, 20 μm.


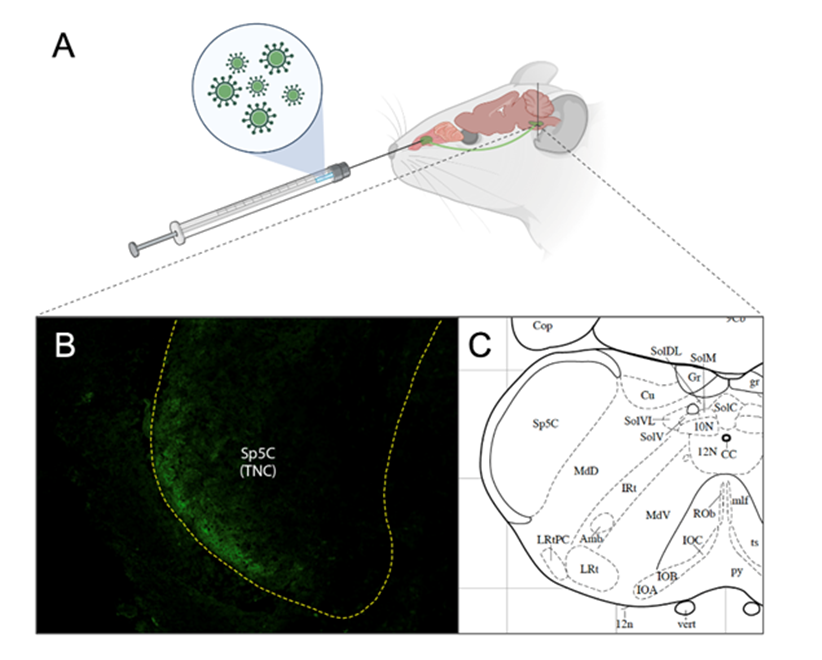


Figure S6. Neurotropic viral tracing in the murine nasal neural pathway. (A) Schematic diagram of neurotropic viral tracing. (B) Fluorescent image of PRV-EGFP-labeled TNC neurons. (C) Mouse brain atlas.


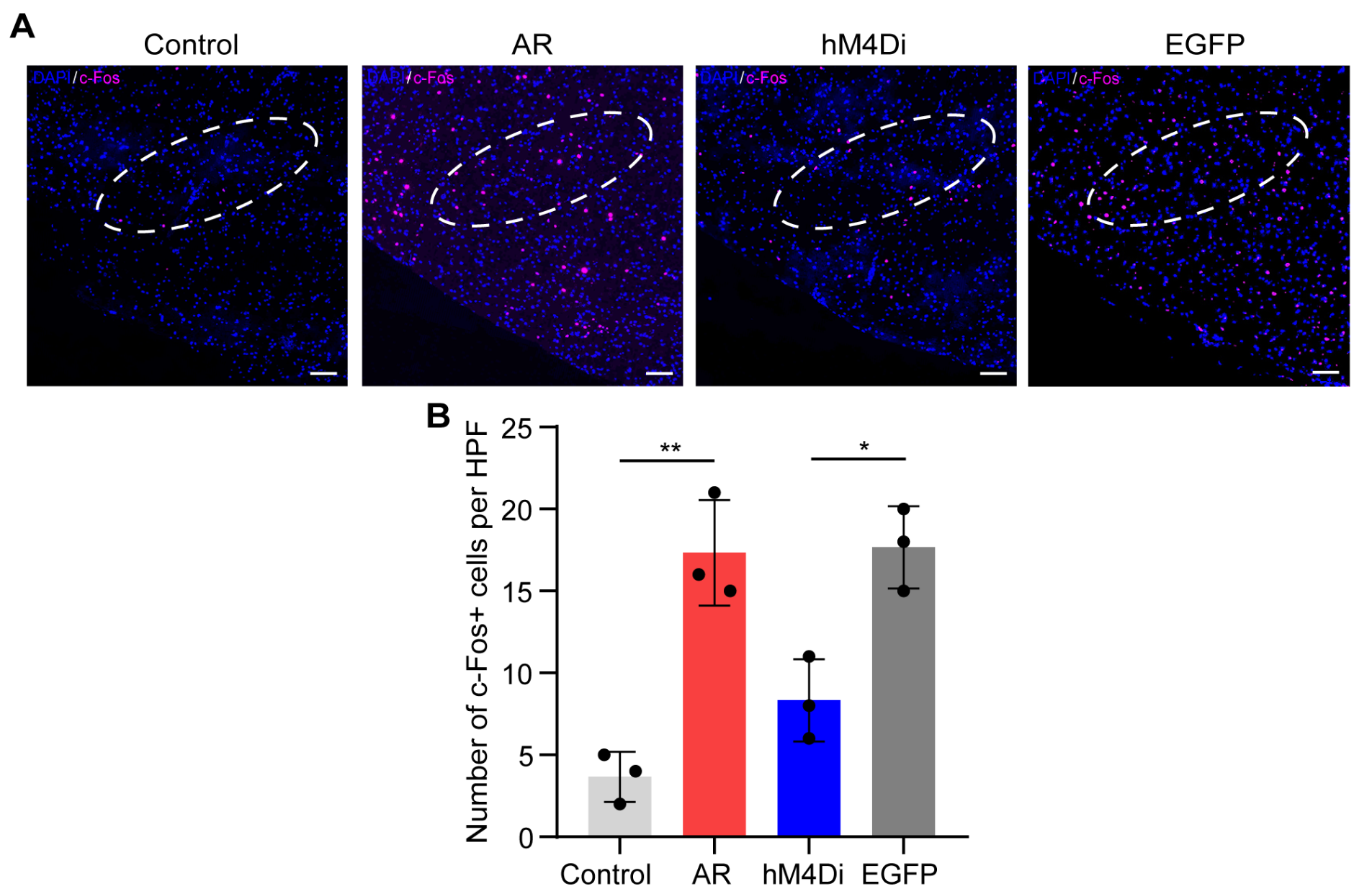


Figure S7. Neuronal activation in the SSN was associated with TNC activity in AR Mice. (A) Immunofluorescence staining for c-Fos. (B) Quantification of c-Fos^+^ cells in the SSN. N = 3 mice per group. Scale bars, 100 μm. *p < 0.05, **p < 0.01.
