# Supplementary material for: Erratum to “Interleukin-4-Mediated NLRP3 Inflammasome Activation in Microglia Contributes to Allergic Rhinitis via Central Sensitization”
Source: Research (Wash D C). 2026 Jan 14;9:1066. doi: 10.34133/research.1066 (PMC12799906; doi:10.34133/research.1066)
Supplement: Supplementary 1 — Fig. S7 [file research.1066.f1.docx]

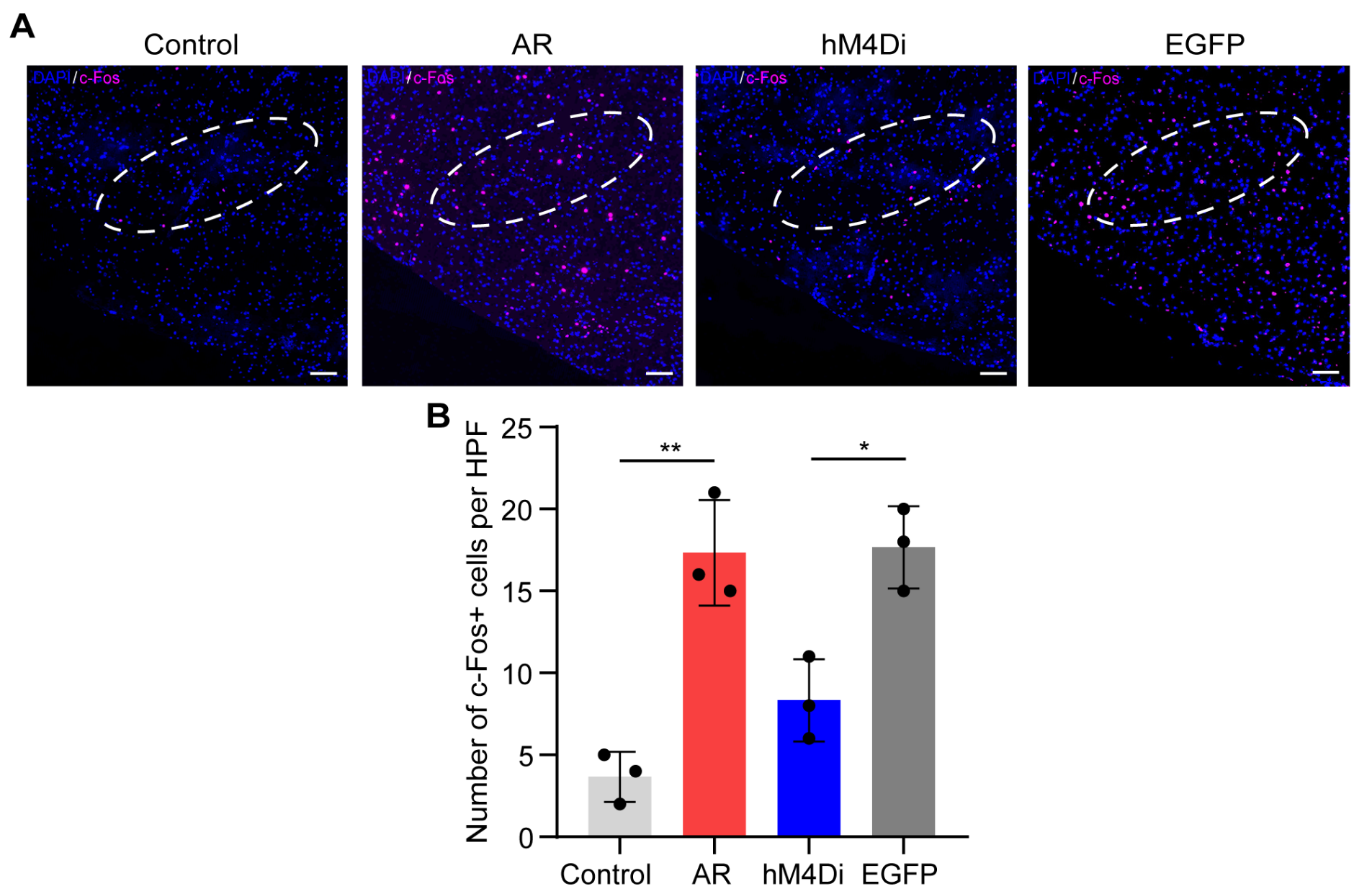


Figure S7. Neuronal activation in the SSN was associated with TNC activity in AR Mice. (A) Immunofluorescence staining for c-Fos. (B) Quantification of c-Fos^+^ cells in the SSN. N = 3 mice per group. Scale bars, 100 μm. *p < 0.05, **p < 0.01.
